# HDAC3 drives type II alveolar epithelial barrier dysfunction via MKP1 deacetylation in radiation‐induced lung injury

**DOI:** 10.1002/ctm2.70877

**Published:** 2026-11-09

**Authors:** Boyang Li, Ning Li, Rui Xiong, Yifan Zuo, Yi Liu, Guoqing Luo, Wenjun Zhang, Zhuya Xiao, Long Wu, Min Peng

**Affiliations:** ^1^ Department of Oncology Renmin Hospital of Wuhan University Wuhan China; ^2^ Central Laboratory Renmin Hospital of Wuhan University Wuhan China; ^3^ Key Laboratory of Hubei Province for Digestive System Disease Renmin Hospital of Wuhan University Wuhan China; ^4^ Department of Thoracic Surgery Renmin Hospital of Wuhan University Wuhan China

**Keywords:** acetylation, apoptosis, HDAC3, MKP1, RILI

## Abstract

Histone deacetylase 3 (HDAC3) is a key biological macromolecule involved in protein acetylation and inflammatory signalling. Although its biological functions are well recognised, the molecular mechanisms through which HDAC3 regulates stress‐induced responses of alveolar epithelial cells during early‐stage radiation‐induced lung injury (RILI) remain incompletely understood. In this study, using AT2‐specific HDAC3 conditional knockout mice as well as primary mouse AT2 and A549 cell models, we found that radiation markedly upregulates HDAC3 expression, whereas HDAC3 knockdown alleviates radiation‐induced disruption of the alveolar epithelial barrier. Transcriptomic analysis further revealed that HDAC3 influences barrier integrity by modulating MAPK‐mediated inflammation and apoptosis. Pharmacological intervention with the selective HDAC3 inhibitor RGFP966 also demonstrated that HDAC3 inhibition mitigates radiation‐induced oxidative stress imbalance. Mechanistically, HDAC3 deacetylates MKP1, enhancing p38 mitogen‐activated protein kinase (p38 MAPK) phosphorylation, promoting FBJ murine osteosarcoma viral oncogene homolog (c‐Fos) expression, and activating activator protein‐1 (AP‐1), thereby driving downstream inflammatory signalling. Collectively, our findings support a role for the HDAC3–MKP1–p38 MAPK–c‐Fos/AP‐1 axis in regulating inflammatory responses in AT2 cells and highlight HDAC3 as a potential molecular target for mitigating alveolar epithelial injury in early‐stage RILI.

## Graphical abstract

HDAC3 promotes radiation‐induced lung injury by deacetylating MKP1 and activating the p38/c‐Fos/AP‐1 signalling axis, leading to excessive inflammatory responses and alveolar epithelial barrier dysfunction. Targeting HDAC3 with genetic deletion or RGFP966‐mediated inhibition effectively alleviates lung injury, supporting HDAC3 as a promising therapeutic strategy for radiation‐induced lung injury.

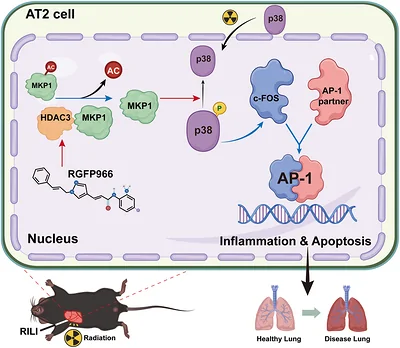

## 1 INTRODUCTION

Cancer remains an important socioeconomic and public health challenge in the 21st century.^1^ Worldwide, cancer accounted for approximately 20 million newly diagnosed cases and 9.7 million deaths in 2022.^1^ Lung and breast cancers are the most commonly diagnosed malignancies in men and women, respectively, accounting for approximately 2.48 million and 2.31 million new cases and imposing a substantial disease burden.^1^ Radiotherapy remains an important treatment modality for both lung and breast cancers; for patients with unresectable stage III non‐small cell lung cancer (NSCLC), concurrent chemoradiotherapy is routinely used as a standard therapeutic approach,^2^ while modern radiotherapy reduces the long‐term risk of breast cancer recurrence and remains an important component of treatment after breast‐conserving surgery.^3^, ^4^

However, ionising radiation (IR) inevitably damages adjacent normal lung tissue while targeting tumours.^5^ Thoracic radiotherapy can be complicated by radiation‐induced lung injury (RILI), a major dose‐limiting adverse effect. Early radiation pneumonitis (RP), an early manifestation of RILI, generally develops within several months after radiotherapy, with approximately 5%–20% of patients developing clinical manifestations, and a proportion of affected patients subsequently progressing to irreversible late‐stage radiation‐induced pulmonary fibrosis (RIPF).^6^, ^7^ Early‐stage RILI is characterised primarily by alveolar epithelial and capillary endothelial injury and may involve DNA damage, oxidative stress, and inflammatory responses, ultimately leading to alveolar epithelial cell apoptosis and impaired barrier integrity.^6^, ^8^, ^9^, ^10^, ^11^, ^12^, ^13^ Inflammation and alveolar epithelial barrier injury are considered important pathological features of early‐stage RILI, and their persistence may further contribute to structural lung damage and fibrotic progression.^14^, ^15^ Therefore, elucidating the molecular mechanisms underlying early‐stage RILI, particularly alveolar epithelial injury and associated inflammatory responses, and identifying potential targets for early intervention may help limit RILI progression.

In healthy lung tissue, type I alveolar epithelial cells constitute the majority of alveolar epithelial cells, whereas type II alveolar epithelial cells (AT2) comprise approximately 15% of the alveolar cell population. AT2 cells, which serve as progenitors capable of generating type I alveolar epithelial cells, have long been regarded as alveolar progenitors and play critical roles in various lung diseases.^16^ Recent single‐cell transcriptomic analyses have revealed that AT2 cells play important roles in mitigating both acute and chronic forms of RILI.^17^ Furthermore, emerging studies demonstrate that targeting AT2 cells to remodel the pulmonary immune regulatory network can significantly alleviate RILI.^18^ Therefore, AT2 cells represent a key cellular focus for exploring the pathogenesis of RILI and potential therapeutic targets. The mammalian histone deacetylase (HDAC) family comprises 18 isoforms classified into four major classes.^19^ Histone deacetylase 3 (HDAC3), a class I histone deacetylase,^20^ in addition to its classical function of histone deacetylation for epigenetic regulation, also acts on non‐histone substrates.^21^ We previously found that inhibition of HDAC3 in AT2 cells restores mitochondrial quality control, significantly reducing apoptotic death of alveolar epithelial cells and preserving alveolar epithelial barrier function in models of acute lung injury (ALI).^22^ Moreover, independent studies have shown that HDAC3 exacerbates pulmonary fibrosis progression by upregulating fibrotic pathways^23^ and enhancing oxidative stress.^24^ HDAC3 has also been implicated in the regulation of pulmonary inflammation.^25^ However, the impact and regulatory mechanisms of HDAC3 in RILI remain unclear and warrant additional study.

Taken together, these findings led us to hypothesise that HDAC3 may contribute to alveolar epithelial barrier dysfunction during early‐stage RILI by regulating signalling pathways associated with inflammation and apoptosis. Therefore, this study focused on early pathological changes in RILI and sought to define how HDAC3 affects AT2 cells and the molecular basis underlying these effects. We found that HDAC3 promoted p38/c‐Fos signalling activity by regulating MKP1 acetylation and was associated with inflammatory responses, apoptosis, and alveolar epithelial barrier dysfunction; pharmacological inhibition of HDAC3 alleviated radiation‐induced lung injury. Collectively, our results support further investigation of HDAC3‐targeted intervention for early‐stage RILI.

## 2 MATERIALS AND EXPERIMENTAL METHODS

### 2.1 Animals

The Animal Care and Use Committee of Renmin Hospital of Wuhan University approved all animal procedures, which were conducted in accordance with the National Institutes of Health (NIH) guidelines for the care and use of laboratory animals. Mice were housed in a specific pathogen‐free (SPF) facility under controlled temperature and humidity, with free access to water and food. Male wild‐type (WT) C57BL/6 mice were obtained from SLAC Laboratory Animal Co., Ltd. (Shanghai, China).

AT2 cell‐specific HDAC3 conditional knockout mice carrying the *Sftpc*‐CreERT2 transgene and homozygous floxed HDAC3 alleles, together with their Cre‐negative littermate controls carrying the same floxed HDAC3 alleles, were derived from an in‐house breeding colony. Breeding and induction procedures were adapted from previously published protocols.^20^ Male mice aged 8–10 weeks received tamoxifen (#HY‐13757A, MedChemExpress, Shanghai, China) intraperitoneally (10 mg/kg, once daily for 5 consecutive days) to induce HDAC3 deletion in AT2 cells. Solvent and injection volumes were prepared according to standard laboratory procedures. Mice treated with RGFP966 received subcutaneous injections of the HDAC3 inhibitor RGFP966 (#HY‐13909, MedChemExpress, Shanghai, China) at 10 mg/kg every other day, starting 7 days before irradiation and continuing thereafter until tissue collection. RGFP966 was prepared using a vehicle composed of 45% saline, 40% PEG300, 10% DMSO, and 5% Tween‐80.

### 2.2 Establishment of the RILI mouse model

The RILI model was established as follows. Mice were placed in a custom‐designed positioning device under isoflurane anaesthesia. The right lung was exposed to a single localised dose of 20 Gy delivered by a 6‐MV X‐ray linear accelerator (Elekta Axesse) with a source‐to‐axis distance (SAD) of 100 cm. A 5‐mm lead‐alloy collimator with an 8 mm × 14 mm rectangular aperture mounted in the gantry was used to deliver a targeted beam precisely to the right lung. Non‐target organs were shielded with lead to ensure that only the thoracic region received radiation. Sham‐irradiated control mice underwent the same anaesthesia, positioning, and immobilisation procedures without radiation exposure.

To minimise bias, animals were allocated to the experimental groups using a randomisation procedure, while histological and molecular analyses were performed by investigators blinded to group allocation. Mice were euthanised at pre‐specified time points (some mice underwent Evans blue assay prior to tissue collection; details are provided below), and lung tissues were harvested (see figure legends or Results for exact time points). Lung tissues were collected and processed according to the downstream applications. Samples designated for RNA and protein analyses were preserved under appropriate conditions, whereas separate specimens were fixed and processed for paraffin embedding and subsequent histological or immunofluorescence (IF) staining. The study followed protocols authorised by the institutional animal care and use committee.

Sample sizes and numbers of replicates were determined according to the requirements of each experiment, as specified in the corresponding figure legends and statistical analysis section.

### 2.3 Establishment of in vitro RILI cellular models

#### 2.3.1 Isolation of primary mouse AT2 cells

Primary AT2 cells were obtained from mouse lungs and maintained in culture according to previously published methods.^22^ Briefly, lung tissues were collected from mice, enzymatically digested, and red blood cells and debris were removed. The resulting cell suspension was further purified by sequential filtration and immune‐adhesion to obtain AT2 cells. Cells were maintained in Ham's F–12K medium (#PM150910, Procell, Wuhan, China) containing 10% fetal bovine serum (FBS; #F0193, Sigma‐Aldrich, USA) at 37°C under humidified conditions with 5% CO_2_. After 72 h of culture, cell morphology and proliferation were assessed before use in subsequent experiments. The 72‐h culture period was chosen to ensure that AT2 cells maintained their original phenotype and function in vitro, avoiding differentiation or functional decline associated with prolonged culture.

##### 2.3.1.1 Cell culture

A549 human lung adenocarcinoma cells were obtained from laboratory stock, while MLE‐12 mouse lung epithelial cells were purchased from Procell (#CL‐0680, Procell, Wuhan, China). Both cell lines were recovered from cryopreserved stocks and routinely cultured under standard conditions at 37°C in a humidified atmosphere containing 5% CO_2_. A549 cells were maintained in Ham's F–12K medium supplemented with 10% FBS and 1% penicillin/streptomycin (#PB180120, Procell, Wuhan, China), whereas MLE‐12 cells were cultured in DMEM/F12 medium (#PM150312, Procell, Wuhan, China) containing 2% FBS, 1% penicillin/streptomycin, 10 nM hydrocortisone, 10 nM β‐estradiol, and 0.5% ITS‐G (100×, #PB180429, Procell, Wuhan, China). Both cell lines were maintained as adherent cultures and routinely passaged using 0.25% trypsin‐EDTA (#PB180226, Procell, Wuhan, China) before reseeding into fresh culture flasks.

Primary human type II alveolar epithelial (AT2) cells were obtained from Procell (#CP‐H209, Procell, Wuhan, China). Cells were maintained in human type II alveolar epithelial cell complete medium (#CM‐H209, Procell, Wuhan, China), which contains FBS, growth supplements, penicillin, and streptomycin, with incubation at 37°C under humidified conditions with 5% CO_2_. Culture vessels were coated with rat‐tail collagen type I (#PB180644, Procell, Wuhan, China) at 2–5 µg/cm^2^ to enhance cell attachment. Following treatment with 0.25% trypsin, cells were passaged at a 1:2 ratio into collagen‐coated culture vessels. Cells were used for subsequent experiments after sufficient attachment and were generally used within 2–3 passages.

##### 2.3.1.2 Cell transfection

A549 and MLE‐12 cells were seeded in culture vessels 18–24 h before transfection and allowed to reach 70%–80% confluence before transfection. Before transfection, cells were switched to serum‐ and antibiotic‐free culture medium. Transfections were performed using Beyotime Lipo8000™ reagent (#C0533, Beyotime, Shanghai, China). For each well of a six‐well plate, 100 pmol siRNA or 2.5 µg plasmid DNA was combined with 4 µL Lipo8000™ and 125 µL Opti‐MEM® medium (#31985070, Thermo Fisher, Shanghai, China), and the mixture was kept at room temperature for 20 min before transfection. For co‐transfection, transfection complexes were prepared by combining 50 pmol siRNA and 2.5 µg plasmid DNA with 5 µL Lipofectamine® 2000 (#11668019, Thermo Fisher, Shanghai, China) in 200 µL Opti‐MEM®. The mixture was incubated at room temperature for 20 min before use. All siRNAs and plasmid DNA were provided by Qinda Biotechnology Co., Ltd. (Wuhan, China). Tables S3 and S4 list the sequences of the siRNAs and the plasmids used for transfection, respectively. The transfection complexes were introduced into the corresponding culture medium and gently mixed to achieve uniform distribution. After 6 h, the transfection medium was replaced with fresh complete medium, and the cells were maintained at 37°C under humidified conditions with 5% CO_2_. Cells were generally collected 36 h after transfection for subsequent analyses.

##### 2.3.1.3 Dual‐luciferase reporter assay

Cells were plated into 24‐well plates and, upon reaching an appropriate confluence, received co‐transfection with the AP‐1 luciferase reporter plasmid (#11535ES03, Yeasen, Shanghai, China), pGMLR‐TK Renilla luciferase reporter plasmid (#11557ES03, Yeasen, Shanghai, China), and the indicated expression plasmids or siRNAs using Lipofectamine 8000 Transfection Reagent. The plasmids utilised for this assay are listed in Table S4. At the designated time point after transfection, cells were collected and lysed using the lysis buffer provided with the Dual Luciferase Reporter Gene Assay Kit (#11402ES60, Yeasen, Shanghai, China). Luciferase activity was assessed for both Firefly and Renilla reporters following the manufacturer's recommended procedure. Firefly luciferase activity was normalised to Renilla luciferase activity, which served as the internal reference, and the relative luciferase activity was calculated accordingly.

#### 2.3.2 Establishment of RILI cellular models

To establish an in vitro model of radiation‐induced pneumonitis, primary mouse AT2 cells, A549 cells, and MLE‐12 cells were cultured under standard conditions before irradiation. The culture medium was changed before irradiation to maintain consistent cellular conditions. A single X‐ray dose was delivered to the cells at room temperature using the same Elekta Axesse linear accelerator as that used for the animal experiments. Cells assigned to the non‐irradiated control group underwent the same procedures but received no radiation treatment. Twenty‐four hours after irradiation, the culture medium was refreshed, and the cells remained in culture until the designated time points, after which they were harvested for downstream analyses, including protein, RNA, and immunofluorescence studies.

### 2.4 Histological and immunofluorescence analyses of lung tissues

The middle or upper lobe of the right lung was fixed by perfusion with 4% paraformaldehyde (#G1101‐500ML, Servicebio, Wuhan, China). The tissues were then embedded in paraffin and sectioned at a thickness of 4 µm, followed by deparaffinisation with xylene and rehydration through a graded ethanol series. To evaluate pathological injury in lung tissues, followed by hematoxylin and eosin (H&E) staining, and the degree of alveolar septal thickening, pulmonary oedema, and infiltration of inflammatory cells was scored under blinded conditions using Image‐Pro Plus 6.0 software according to previously reported criteria.^26^ For immunofluorescence staining, antigen retrieval was performed on the paraffin sections. The sections were incubated with the primary antibodies specified in Table S1. After washing, the sections were incubated with a Cy3‐conjugated secondary antibody (#AS007, ABclonal, Wuhan, China) and FITC‐conjugated secondary antibody (#AS011, ABclonal, Wuhan, China), followed by nuclear counterstaining with DAPI (#C1002, Beyotime, Shanghai, China). Fluorescence images were acquired on an Olympus fluorescence microscope (Tokyo, Japan), and the fluorescence‐positive area was quantified semi‐quantitatively using Image‐Pro Plus 6.0 software.

### 2.5 Protein expression and interaction analysis

#### 2.5.1 Western blot analysis

Lung tissues and cell samples were subjected to protein extraction with RIPA lysis buffer (#P0013B, Beyotime, Shanghai, China), followed by protein quantification using the BCA assay kit (#P0398S, Beyotime, Shanghai, China). For each sample, 30 µg of total protein was loaded onto 8%–12% SDS‐PAGE gels for electrophoretic separation, followed by electrotransfer onto 0.45 µm PVDF membranes (#10600023, Servicebio, Wuhan, China). Following protein transfer, the membranes were blocked in 5% skim milk powder (#GC310001‐100 g, Seville, Wuhan, China) and incubated overnight at 4°C with the primary antibodies. After washing, the membranes were exposed to HRP‐conjugated goat anti‐rabbit secondary antibodies for 1 h at room temperature. Chemiluminescent signals were developed using an ECL detection kit (#P0018S, Beyotime, Shanghai, China), and the resulting signals were recorded for subsequent analysis. Band intensities were subsequently quantified using Image Lab software (BIO‐RAD Laboratories, Inc.). The primary antibodies used in this study are listed in Table S1.

#### 2.5.2 Co‐immunoprecipitation analysis

A549, MLE‐12, and primary human type II alveolar epithelial (AT2) cells were lysed in IP lysis buffer (#P0013, Beyotime, Shanghai, China) at 4°C for 30 min, followed by centrifugation at 13 000 rpm for 10 min to collect the supernatant. Protein A/G magnetic beads (#88803, Thermo, Shanghai, China) were pre‐incubated with 1–5 µg of the specific antibody at 4°C for 1–2 h to form the antibody–bead complex. For immunoprecipitation, the pre‐assembled antibody–bead complexes were transferred into the protein supernatant and incubated at 4°C overnight (≥12 h). The resulting immune complexes were subjected to 4–6 washing cycles with 500 µL IP wash buffer (20 mM Tris‐HCl, pH 7.4; 150 mM NaCl; 0.5% NP‐40) and subsequently eluted in Laemmli sample buffer supplemented with β‐mercaptoethanol. The eluates were heated at 100°C for 10 min before analysis. SDS‐PAGE was subsequently performed, and the subsequent Western blot procedures were carried out as described above. The interaction strength of different proteins was semi‐quantified based on immunoblotting signal intensity. For Co‐IP analysis of nuclear and cytoplasmic fractions, the two cellular compartments were separated using the NE‐PER Nuclear and Cytoplasmic Extraction Kit (#78833, Thermo Scientific, Shanghai, China). The Co‐IP procedure was then carried out as described above.

#### 2.5.3 GST‐pull down analysis

GST‐HDAC3 and His‐MKP1 recombinant proteins were expressed using the corresponding plasmids listed in Table S4 and purified using BeyoGold™ GST‐tag Purification Resin (#P2250, Beyotime, Shanghai, China) and BeyoGold™ His‐tag Purification Resin (#P2218, Beyotime, Shanghai, China), respectively. All recombinant proteins were expressed in BL21(DE3) competent cells (#C504‐02, Vazyme, Nanjing, China) following induction with isopropyl β‐D‐1‐thiogalactopyranoside (IPTG; #ST099‐5 g, Beyotime, Shanghai, China), followed by bacterial cell lysis. Before the pull‐down assay, a small aliquot of each purified protein mixture was collected as the Input, and the remaining samples were used for the subsequent GST pull‐down assay. Purified GST‐HDAC3 or GST protein was incubated with BeyoMag™ Anti‐GST Magnetic Beads (#P2138‐0.5 mL, Beyotime, Shanghai, China) to capture the GST‐tagged proteins. Purified His‐MKP1 was introduced into the bead‐bound protein preparation and incubated at 4°C, with GST plus His‐MKP1 serving as the negative control. Following incubation, the beads were recovered by magnetic separation and subjected to washing. The associated proteins were subsequently released with an acidic elution buffer (0.1 M Glycine‐HCl, pH 3.0) and immediately brought to neutral pH using neutralisation buffer (0.5 M Tris‐HCl, pH 7.4, 1.5 M NaCl). The eluates were mixed with SDS‐PAGE sample loading buffer and subjected to denaturation, followed by SDS‐PAGE and Western blotting. Anti‐GST and anti‐His antibodies were used to detect the corresponding proteins.

### 2.6 Relative quantification of gene expression by RT‐PCR

Lung tissue and cellular samples were subjected to total RNA extraction with TRIzol reagent. RNA integrity and purity was assessed with a NanoDrop ND‐1000 spectrophotometer (Invitrogen, Shanghai, China). For cDNA synthesis, 1 µg of RNA was subjected to reverse transcription using the reverse transcription kit (#RK20400, ABclonal, Wuhan, China). Quantitative PCR was performed using the qPCR reaction mix (#RK21203, ABclonal, Wuhan, China) on a CFX Connect real‐time PCR system (Bio‐Rad, Hercules, CA, USA). GAPDH served as the reference gene for normalisation, and relative transcript levels were determined by the 2^–ΔΔCt^ method. The primer sequences used for qPCR are provided in Table S2.

### 2.7 Construction and amplification of overexpression plasmid vectors

A full‐length human *HDAC3* coding sequence (NM_003883.4), synthesised by Qinda Biotechnology Co., Ltd. (Wuhan, China), was inserted into the EcoRI/ApaI sites of the pCMV‐3×FLAG‐Neo vector to generate a construct bearing a C‐terminal 3×FLAG tag. The required point mutants (Y298F, N89A, K137A, Y182A, N197A, E212A) were generated in the same vector backbone by the company. The MKP1 K57R mutant was constructed in the pcDNA3.1(+) expression vector. All sequences were verified by Sanger sequencing, plasmids listed in Table S4. Each recombinant plasmid was transformed into competent DH5α E. coli and plated on LB agar containing 100 µg/mL ampicillin, followed by overnight incubation at 37°C. Individual colonies were transferred to 100 mL LB‐Amp broth for high‐copy plasmids or 200 mL for low‐copy plasmids and grown at 37°C with agitation at 220 rpm for 12–16 h, reaching an A600 of approximately 2.0–4.0. Plasmid purification was performed using the endotoxin‐free maxi plasmid DNA purification kit (#DP117, TIANGEN Biotechnology, Beijing, China). Bacterial cultures were harvested, lysed, neutralised, filtered, loaded onto the column, washed, and eluted with TB buffer at 65°C. The purified plasmid DNA was assessed for purity based on an A260/A280 ratio of 1.8–2.0 and stored at −20°C until further use.

### 2.8 Transcriptomic and bioinformatic analysis

#### 2.8.1 GEO single‐gene analysis

Publicly available gene expression datasets, including GSE70943, GSE41789, and GSE242706, were retrieved through the Gene Expression Omnibus (GEO) database and analysed using the GEO2R web‐based analysis tool. For the microarray datasets GSE70943 and GSE41789, expression data were subjected to log2 transformation when appropriate and analysed with differential expression analysis performed in limma using linear modelling and empirical Bayes adjustment. For the RNA‐sequencing dataset GSE242706, raw count data were normalised using the DESeq2 size‐factor method with positive‐count‐based estimation (sfType = ‘poscount’) and subjected to the DESeq2 Wald test. Multiple testing was addressed by controlling the false discovery rate (FDR). GSE70943 included 5 control and 27 irradiated samples, GSE41789 included 3 control and 3 irradiated samples, and GSE242706 included 4 control and 4 irradiated samples. Statistical significance was defined as an FDR‐adjusted *p* value below  .05.

#### 2.8.2 RNA‐seq differential expression analysis (simplified version)

Total RNA was extracted from primary mouse AT2 cells using TRIzol reagent (#5596‐026, Invitrogen, Shanghai, China). RNA quality was assessed with a NanoDrop ND‐1000 spectrophotometer (Invitrogen, Shanghai, China) using a NanoDrop ND‐1000 spectrophotometer (Invitrogen, Shanghai, China) together with an Agilent 2100 Bioanalyzer (Agilent, CA, USA). RNA libraries were generated with the NEBNext® Ultra™ RNA Library Prep Kit and subjected to paired‐end sequencing (150 bp per read; PE150) on an Illumina NovaSeq™ 6000 platform. Three biological replicates were included in each group (*n* = 3), with a sequencing depth of approximately 20 million read pairs per sample. Raw sequencing data were processed by removing adapter contamination, low‐quality reads, and reads containing N bases, yielding the clean‐read dataset used for subsequent analyses. The resulting reads were mapped to the reference genome with Bowtie, and gene‐level read counts were quantified using HTSeq. Differentially expressed genes were identified with edgeR, applying thresholds of FDR < 0.05 and |fold change| ≥ 1.2. Functional enrichment of the resulting DEGs was subsequently assessed using the clusterProfiler package, including Gene Ontology (GO) and Kyoto Encyclopedia of Genes and Genomes (KEGG) analyses. For these enrichment analyses, statistical significance was determined using a Benjamini–Hochberg‐adjusted *p* value threshold of 0.05. To investigate protein‐level associations among the identified genes, a protein–protein interaction (PPI) network was constructed using the STRING database with an interaction score cutoff of > 0.15, and the resulting network was visualised in Cytoscape.

### 2.9 Evans blue staining

Pulmonary alveolar epithelial barrier permeability was assessed by relative Evans blue quantification. At the end of radiation exposure, 2% Evans blue dye (#ST3273‐5 g, Beyotime, Shanghai, China) was administered intravenously through the tail vein at a dose of 2 mL/kg. Five minutes later, mice were euthanised, and the right lungs were collected for whole‐lung imaging to visualise dye distribution. Subsequently, the left lung hilum was exposed, and bronchoalveolar lavage fluid (BALF) and serum were collected. The absorbance signal was recorded at 620 nm with a PerkinElmer EnSight microplate reader, and data were analysed with Kaleido 2.0 software to calculate the BALF/serum ratio for relative quantification.

### 2.10 TUNEL staining

Cell apoptosis was evaluated by TUNEL staining, with DAPI used for nuclear counterstaining. For cell samples, fixation and permeabilisation were performed before TUNEL staining. Lung tissue sections were processed without an additional fixation step. The TUNEL reaction was carried out using the TUNEL Apoptosis Kit (#G1504‐50T, Servicebio, Wuhan, China) in accordance with the manufacturer's instructions, and fluorescence signals were examined using a fluorescence microscope.

### 2.11 Oxidative stress assessment

Catalase (CAT) activity was quantified colorimetrically in lung tissues and cells using a Catalase Assay Kit (#S0051, Beyotime, Shanghai, China). NADPH oxidase activity was measured using a colorimetric assay kit (#A127‐1‐1) according to the instructions provided with the kit. Dihydroethidium (DHE; #G1746‐100T, Servicebio, Wuhan, China) fluorescence was used to assess reactive oxygen species (ROS) levels in lung tissue and cell samples. Nuclear counterstaining was subsequently performed with Hoechst 33258 (#G1011‐10ML, Servicebio, Wuhan, China), and fluorescence was visualised using a fluorescence microscope.

### 2.12 Survival rate, body weight index, and lung dry/wet weight ratio

Survival rate was recorded over a 28‐day period after right lung irradiation to assess the impact of HDAC3 deficiency, with food and water provided ad libitum; Kaplan–Meier curves were subsequently generated for survival analysis. The lung dry‐to‐wet weight ratio (D/W) was used as an indicator of pulmonary oedema. At the end of the experimental period, the lungs were collected and weighed before and after drying, and the D/W ratio was calculated accordingly. Body weight was monitored throughout the experimental period, and the body weight index was plotted over time.

### 2.13 Statistical analysis

Data are expressed as mean ± SEM. For two‐group comparisons, the unpaired Student's t‐test or Mann–Whitney U test was selected according to data distribution. For analyses involving multiple groups or variables, one‐way or two‐way ANOVA was used as appropriate, with the Kruskal–Wallis test applied when parametric assumptions were not satisfied. For experiments with a sample size of *n* = 3, non‐parametric tests were used. Statistical significance was defined as *p* < .05, and all analyses were conducted with GraphPad Prism 9. The figure legends specify the number of biological replicates for each group, defined as independent experiments for cell‐based studies and individual mice for in vivo experiments.

## 3 RESULTS

### 3.1 HDAC3 is consistently elevated in early‐stage RILI models in animal studies and cell‐based experiments

To initially assess the association between *HDAC3* and early‐stage RILI, we first examined transcriptomic data deposited in the Gene Expression Omnibus (GEO). The analysis revealed an elevation in *HDAC3* expression after irradiation in lung tissues from both rhesus macaques and mice (Figure 1A). In contrast, *HDAC1/2*, which belong to the same HDAC family, did not show apparent radiation‐induced upregulation in the corresponding models (Figure S1A and B), suggesting that the change in *HDAC3* may exhibit a certain degree of selectivity. In addition, *HDAC3* expression was also increased following irradiation in a human lung alveolar chip model (Figure 1A). Together with our previous research and relevant literature reports,^22^, ^24^, ^27^ these findings support a potential involvement of *HDAC3* in radiation‐induced pulmonary injury and provide a rationale for further investigating its role in early‐stage RILI.

**FIGURE 1:**
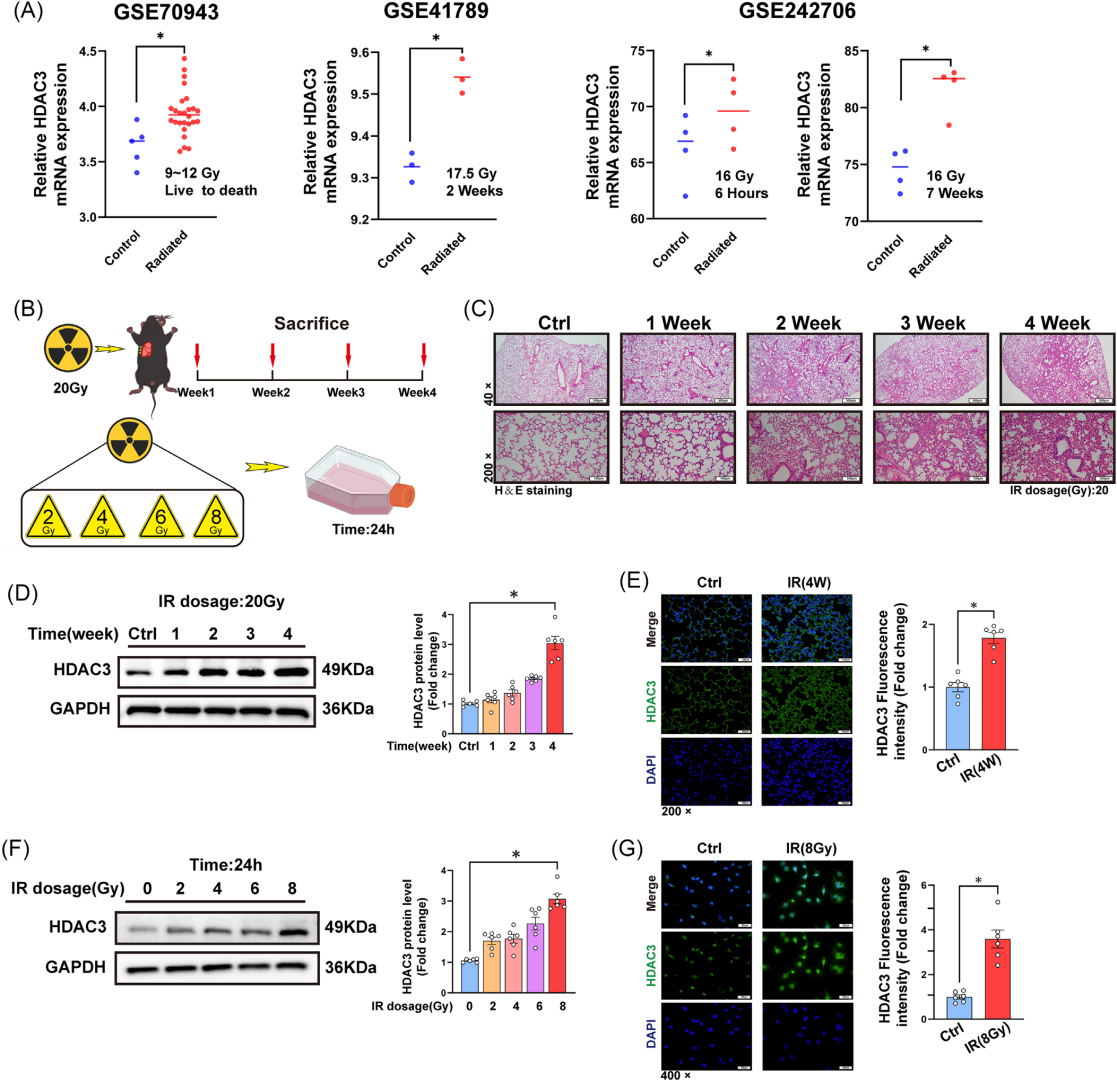
Radiation induces HDAC3 upregulation in lung tissues and alveolar epithelial cells. (A) *HDAC3* expression was analysed using two public GEO datasets. Left: lung tissues from rhesus macaques exposed to 9–12 Gy thoracic irradiation (GSE70943); middle: lung tissues from mice exposed to 17.5 Gy thoracic irradiation (GSE41789); right: a human lung alveolus chip model exposed to 16 Gy irradiation (GSE242706). (B) Schematic illustration of RP models in mice and cells. (C) Representative H&E staining images of mouse lung tissues (*n* = 6). Scale bars, 500 and 100 µm, respectively. (D) Representative Western blot and corresponding quantification of HDAC3 in mouse lung tissues following thoracic irradiation or control treatment (*n* = 6). (E) Representative immunofluorescence staining images of HDAC3 (green) in mouse lung tissues at 0 and 4 weeks and corresponding fluorescence quantification. Blue indicates cell nuclei stained with DAPI (*n* = 6). Scale bars, 100 µm. (F) Representative Western blot and corresponding quantification of HDAC3 in primary mouse AT2 cells isolated from mice, following in vitro irradiation or control treatment (*n* = 6). (G) Representative immunofluorescence staining images of HDAC3 (green) in primary mouse AT2 cells isolated from mice at 0 and 8 Gy and corresponding fluorescence quantification. Blue indicates cell nuclei stained with DAPI (*n* = 6). Scale bars, 50 µm. Differences were assessed by Student's *t* test or two‐way ANOVA, as appropriate. Values are presented as mean ± standard error of the mean (SEM). *p < .05, versus the indicated group; ns, not significant.

To further validate these findings, we established in vivo and in vitro models of early‐stage RILI. To minimise potential interference from radiation‐induced cardiac injury with the evaluation of pulmonary phenotypes, mice received localised irradiation of the right lung at 20 Gy. This dose was selected based on previous studies establishing mouse models of RILI using localised lung irradiation.^28^ Lung tissues were collected at 1, 2, 3, and 4 weeks after irradiation (Figure 1B). In parallel, primary mouse alveolar type II epithelial cells (hereafter referred to as primary mouse AT2 cells) and A549 cells received 8 Gy, while MLE‐12 cells received 10 Gy irradiation in vitro, as previously reported^29^, ^30^, ^31^ (Figure 1B).

In lung tissues exposed to 20 Gy irradiation, H&E staining showed progressively increased inflammatory injury during the 0–4 week period, characterised by alveolar septal thickening, inflammatory cell infiltration, and disruption of alveolar architecture (Figure 1C). Western blotting demonstrated a progressive increase in HDAC3 protein levels following irradiation, beginning at week 1 and peaking at week 4 (Figure 1D). Immunofluorescence staining further confirmed this trend (Figure 1E). In primary mouse AT2 cells exposed to 0, 2, 4, and 8 Gy for 24 h, western blotting (Figure 1F) and immunofluorescence (Figure 1G) analysis showed that HDAC3 protein levels increased with increasing radiation doses. Further qPCR analysis demonstrated elevated *HDAC3* mRNA levels in irradiated mouse lung tissues (Figure S2A) and primary mouse AT2 cells (Figure S2B), together with increased expression of *IL‐1β*, *IL‐6*, and *TNF‐α*, key mediators of inflammation (Figure S2A and B).

A549 cells, derived from human lung adenocarcinoma, retain partial AT2‐like characteristics under long‐term culture conditions^32^ and offer advantages including human origin, high transfection efficiency, stability, and broad availability.^33^ They have also been widely used in studies investigating inflammatory mechanisms of RILI.^34^ Therefore, when primary mouse AT2 cells were limited, A549 cells were employed as a complementary model. Following 8 Gy irradiation for 24 h, *HDAC3* and proinflammatory cytokine mRNA levels were synchronously upregulated in A549 cells (Figure S2C). Similarly, in MLE‐12 cells, 10 Gy irradiation followed by 24 h of culture resulted in increased *HDAC3* and proinflammatory cytokine‐related mRNA levels (Figure S2D).

Accordingly, the subsequent in vivo study employed 20 Gy irradiation of the right lung, with lung tissues collected 4 weeks after irradiation. For the in vitro experiments, primary mouse AT2 cells and A549 cells were exposed to 8 Gy irradiation, whereas MLE‐12 cells were exposed to 10 Gy irradiation, followed by analysis at 24 h after irradiation. Collectively, across the radiation models examined in animals and cultured cells from different species, HDAC3 expression was consistently elevated, suggesting that HDAC3 may contribute to early‐stage RILI.

### 3.2 HDAC3 deletion in AT2 cells alleviates disease manifestations of early‐stage RILI

We first generated mice with conditional *HDAC3* deletion in AT2 cells (HDAC3‐CKO) (construction method detailed in our previous study^22^). Briefly, *HDAC3* ^flox/flox^ mice were generated using CRISPR–Cas9 and subsequently bred with *Sftpc*‐CreERT2 transgenic mice to obtain HDAC3‐CKO mice expressing Cre recombinase specifically in AT2 cells (Figure 2A and B). Genotypes were confirmed by PCR (Figure S3A).

**FIGURE 2:**
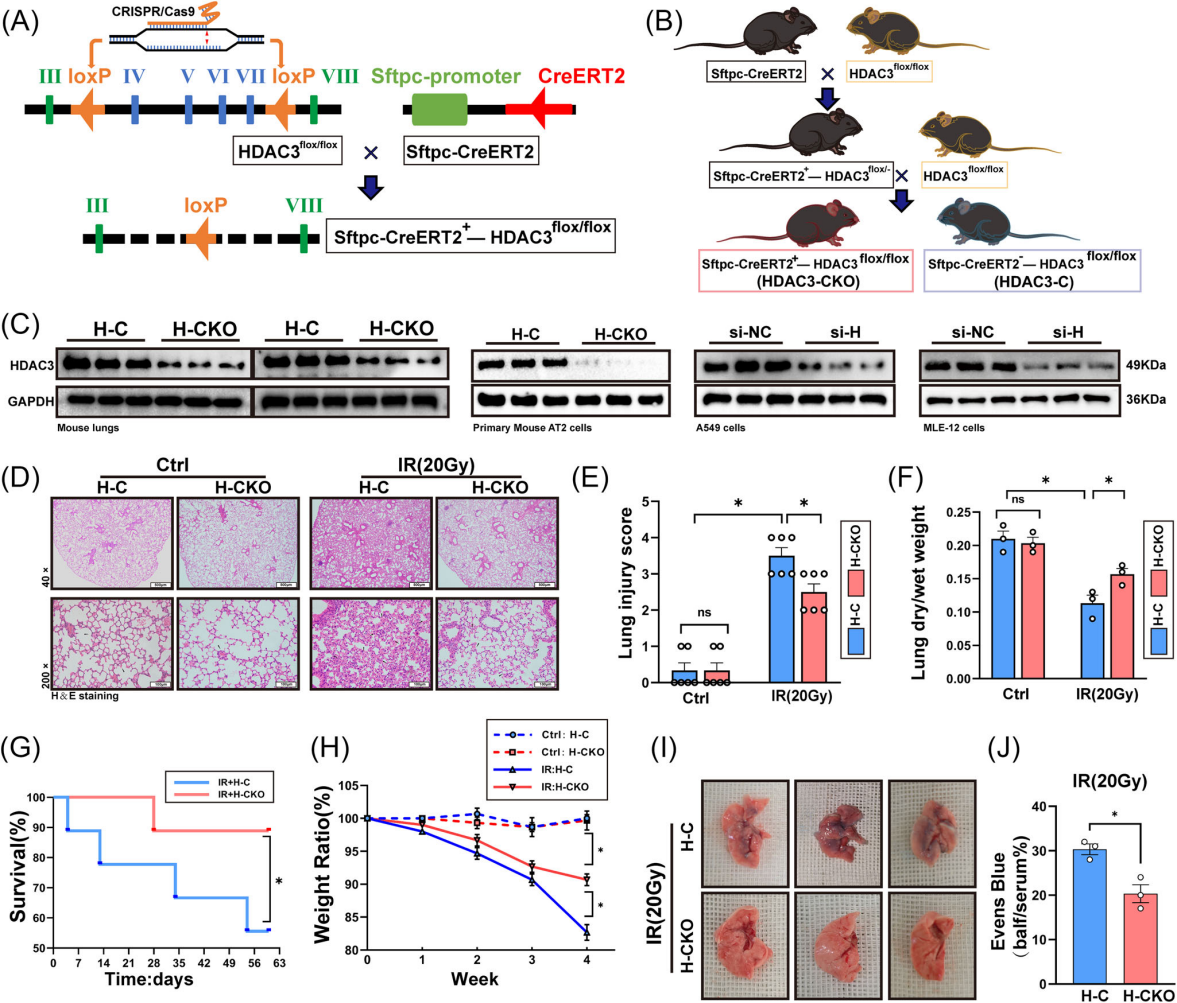
AT2‐specific deletion of *HDAC3* attenuates RILI. (A) Schematic illustration of the generation of *HDAC3* ^flox/flox^ mice by introducing loxP sites into the intronic regions flanking exons 4–7 of the *HDAC3* gene via the CRISPR–Cas9 system, creating a conditional, non‐functional *HDAC3* allele. (B) Schematic illustration of the breeding strategy for generating AT2 cell–specific *HDAC3* conditional knockout (HDAC3‐CKO) mice by crossing *Sftpc*‐CreERT2 transgenic mice with *HDAC3* ^flox/flox^ mice. Prior to thoracic irradiation, Cre recombination in AT2 cells was induced by intraperitoneal administration of tamoxifen (10 mg/kg in corn oil) for 5 consecutive days. (C) Western blot of HDAC3 in mouse lung tissues from HDAC3‐C or HDAC3‐CKO mice (*n* = 6). Representative Western blot of HDAC3 in primary mouse AT2 cells isolated from HDAC3‐C or HDAC3‐CKO mice and in A549 cells and MLE‐12 cells transfected with non‐targeting control siRNA (si‐NTC, hereafter si‐NC) or si‐HDAC3 (*n* = 3). (D–E) Representative H&E staining images of mouse lung tissues and corresponding lung injury scores (*n* = 6). Black arrows indicate representative areas of inflammatory lung injury. Scale bars, 500 and 100 µm, respectively. (F) Ratio of lung dry weight to wet weight at 4 weeks (*n* = 3). (G) Kaplan–Meier survival curves of mice from 1 to 9 weeks (*n* = 10). (H) Body weight index changes of mice from 1 to 4 weeks (*n* = 3). (I, J) Whole‐lung images of mice following Evans blue dye injection (2 mL/kg via tail vein) to assess pulmonary vascular permeability, with corresponding quantification of BALF/serum ratio based on optical density measured at 620 nm (right) (*n* = 3). Differences were assessed by Mann–Whitney U test, Kruskal–Wallis test, two‐way ANOVA, or log‐rank (Mantel–Cox) test, as appropriate. Values are presented as mean ± standard error of the mean (SEM). *p < .05, versus the indicated group; ns, not significant.

Western blot analysis revealed a marked reduction in lung HDAC3 protein expression in HDAC3‐CKO mice compared with HDAC3‐C mice (Figure 2C), accompanied by a similar reduction in *HDAC3* mRNA levels (Figure S3B). Primary mouse AT2 cells isolated from HDAC3‐CKO mice also exhibited significantly decreased HDAC3 protein and mRNA level (Figures 2C and S3C, respectively). In A549 cells, si‐HDAC3 transfection efficiently reduced HDAC3 protein and mRNA levels relative to si‐NC controls (Figures 2C and S3D, respectively). Similarly, in MLE‐12 cells, si‐HDAC3 transfection efficiently reduced HDAC3 protein and mRNA levels relative to si‐NC controls (Figures 2C and S3E, respectively).

H&E staining and lung injury scoring revealed pronounced features of early‐stage RILI in the HDAC3‐C group, including oedema, inflammatory exudates, and alveolar septal thickening, which were substantially alleviated in the HDAC3‐CKO group (Figure 2D and E). At week 4, lung dry‐to‐wet weight ratios were lower in HDAC3‐CKO mice, indicating reduced pulmonary fluid accumulation (Figure 2F). Survival analysis demonstrated higher survival rates in HDAC3‐CKO mice from week 1 to week 9 post‐irradiation (Figure 2G). Body weight monitoring indicated more stable weights in the HDAC3‐CKO group during the observation period (Figure 2H). Evans blue dye was used to assess pulmonary vascular permeability as an indicator of alveolar‐capillary barrier disruption. Evans blue staining showed increased pulmonary permeability in HDAC3‐C mice following irradiation, whereas HDAC3‐CKO mice exhibited reduced permeability (Figure 2I). The BALF/serum optical density ratio was subsequently determined using a microplate reader to further assess pulmonary permeability (Figure 2J).

Collectively, AT2 cell–specific deletion of *HDAC3* attenuates RILI‐associated pathological alterations, supporting its protective role in early‐stage RILI and providing a useful model for subsequent functional and mechanistic studies.

### 3.3 HDAC3 loss preserves alveolar epithelial barrier function and attenuates inflammation in early‐stage RILI

The alveolar epithelial barrier is primarily composed of AT1 cells, which form an ultrathin physiological interface to enable efficient gas exchange, fluid transport, and pathogen defence, whereas AT2 cells contribute to barrier maintenance by secreting surfactant and serve as progenitor cells that repair damaged structures through proliferation and differentiation, thereby contributing substantially to the maintenance of barrier homeostasis and function.^35^ Previous studies have shown that in RILI, alveolar epithelial barrier dysfunction – including disruption of epithelial junctional proteins and increased alveolar–capillary permeability, along with pro‐inflammatory cytokine release, with these processes contributing to the progression of lung injury.^36^

To assess the protective effect of HDAC3 deficiency, we first examined barrier‐related markers and inflammatory cytokines. Immunofluorescence analysis showed markedly reduced ZO‐1, E‐cadherin, and Occludin signals in irradiated HDAC3‐C lung tissues, with discontinuous or fragmented distribution, whereas signal intensity and localisation were largely preserved in HDAC3‐CKO lungs (Figures 3A and B and S4). These differences in protein expression were further verified by Western blotting (Figure 3C). These changes were accompanied by improvements in inflammatory status: HDAC3 deficiency significantly reduced the transcript levels of inflammatory cytokines in irradiated mouse lung tissues (Figure 3D). In primary mouse AT2 cells exposed to irradiation in vitro, HDAC3‐CKO cells similarly retained higher levels of ZO‐1, E‐cadherin, and Occludin (Figure 3E), and showed reduced transcript levels of these pro‐inflammatory cytokines (Figure 3F), supporting a role for HDAC3 in AT2 cell barrier disruption. In A549 cells, irradiation also reduced ZO‐1, E‐cadherin, and Occludin expression, while HDAC3 knockdown significantly restored protein levels, consistent with observations in primary mouse AT2 cells (Figure 3G). Similarly, HDAC3 knockdown reduced transcript levels of these pro‐inflammatory cytokines in irradiated A549 cells (Figure 3H).

**FIGURE 3:**
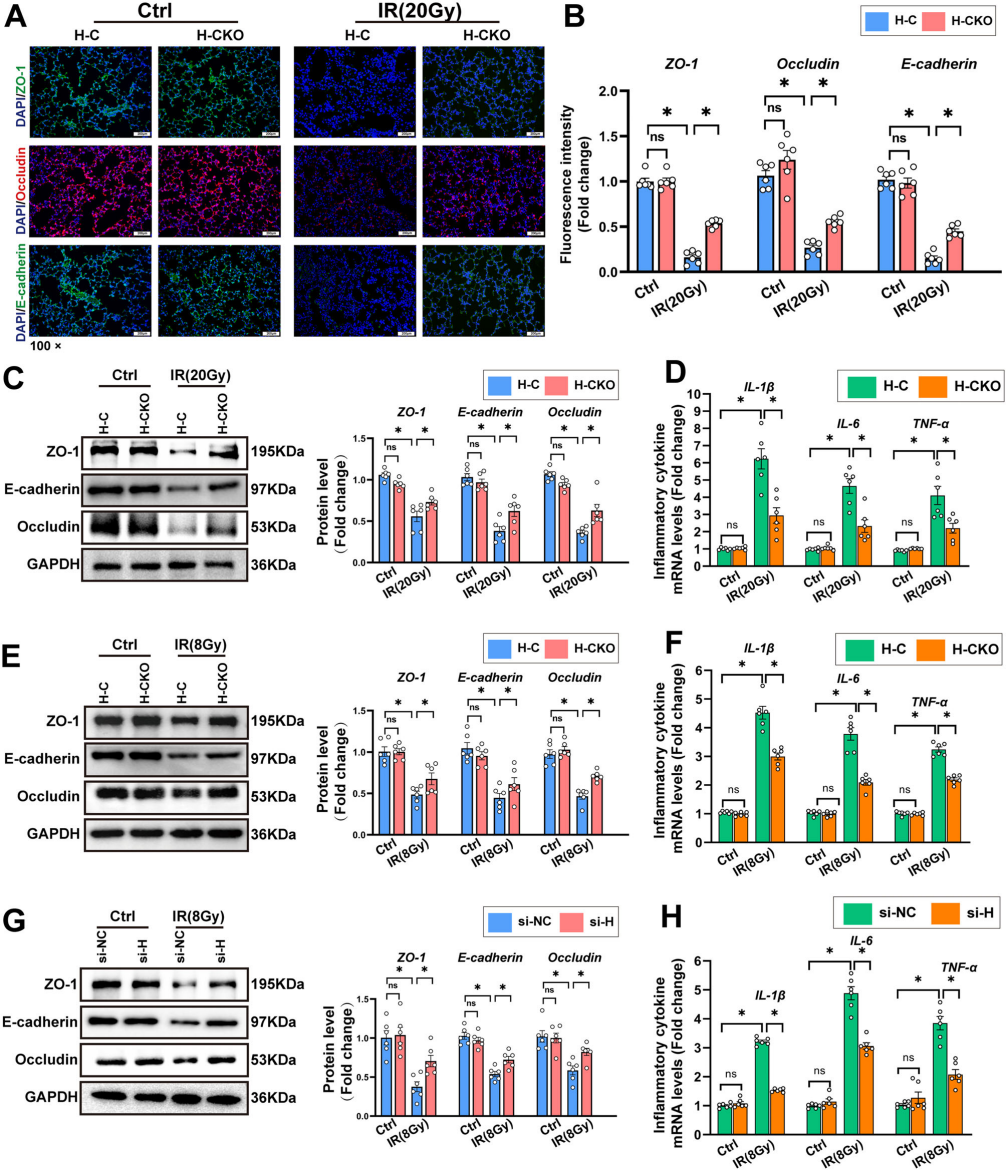
HDAC3 regulates alveolar barrier integrity and inflammatory responses in RILI. (A, B) Representative immunofluorescence staining images of ZO‐1 (green, top), Occludin (red, middle) and E‐cadherin (green, bottom) in mouse lung tissues, with corresponding fluorescence quantification. Blue indicates cell nuclei stained with DAPI (*n* = 6). Scale bars, 200 µm. (C) Representative Western blot and corresponding quantification of ZO‐1, E‐cadherin, and Occludin in mouse lung tissues (*n* = 6). (D) Relative mRNA levels of *IL‐1β*, *IL‐6*, and *TNF‐α* in mouse lung tissues (*n* = 6). (E) Representative Western blot and corresponding quantification of ZO‐1, E‐cadherin, and Occludin in primary mouse AT2 cells (*n* = 6). (F) Relative mRNA levels of *IL‐1β*, *IL‐6*, and *TNF‐α* in primary mouse AT2 cells (*n* = 6). (G) Representative Western blot and corresponding quantification of ZO‐1, E‐cadherin, and Occludin in A549 cells (*n* = 6). (H) Relative mRNA levels of *IL‐1β*, *IL‐6*, and *TNF‐α* in A549 cells (*n* = 6). Differences were assessed by two‐way ANOVA. Values are presented as mean ± standard error of the mean (SEM). *p < .05, versus the indicated group; ns, not significant.

Collectively, results from multiple models demonstrate that HDAC3 deficiency preserves alveolar epithelial barrier integrity and attenuates radiation‐induced inflammation, supporting a potential role for HDAC3 inhibition in early‐stage RILI.

### 3.4 HDAC3 deficiency alleviates alveolar epithelial apoptosis and inflammation via p38‐MAPK/c‐Fos in early‐stage RILI

To investigate the mechanisms by which HDAC3 regulates the pathology and phenotypes of RILI, primary mouse AT2 cells were isolated from conditional *HDAC3* knockout mice and subjected to RNA sequencing (RNA‐seq) following irradiation exposure. Among the 55 868 genes analysed, 1081 showed differential expression between the HDAC3‐CKO and HDAC3‐C groups (Figure S5A). Functional annotation indicated that several of these differentially expressed genes (DEGs) were closely associated with inflammatory processes (Figure S5B). Further Gene Ontology (GO) analysis (Figure 4A) revealed that these DEGs were significantly enriched in cytokine‐mediated signalling pathways and associated with epithelial cell apoptotic processes, suggesting that inflammatory signalling and epithelial apoptosis may jointly contribute to the observed pathological phenotypes.

**FIGURE 4:**
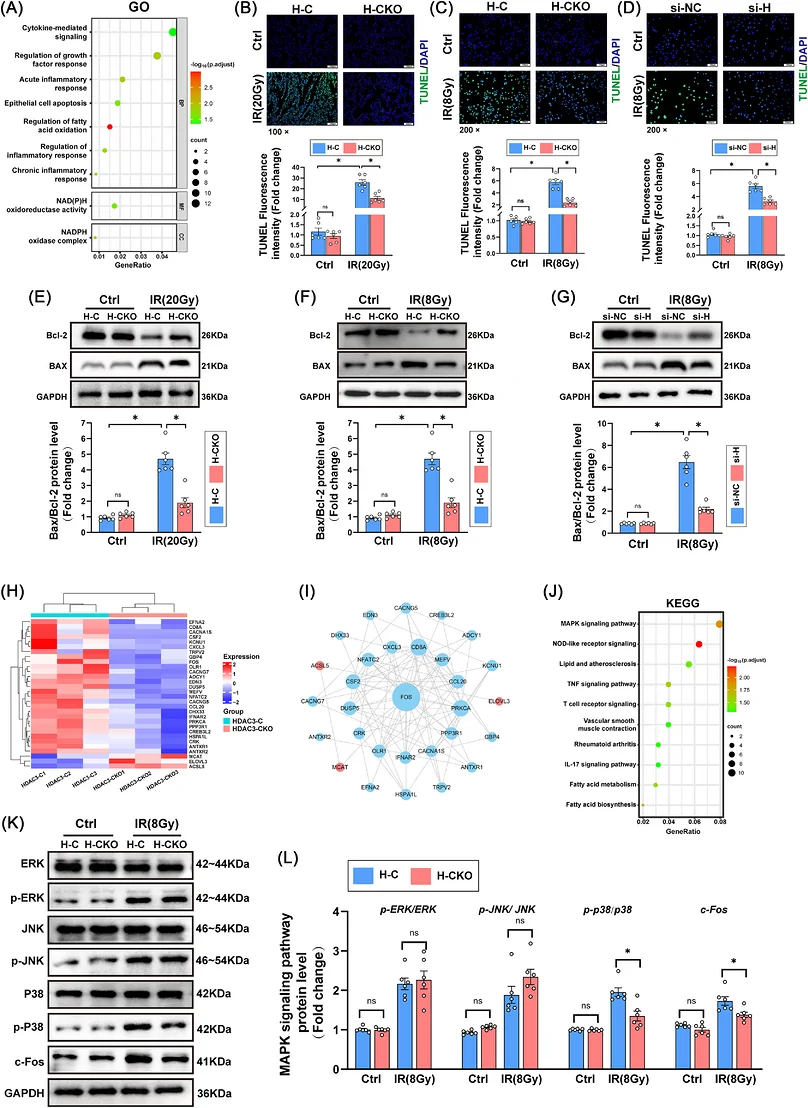
HDAC3 deficiency suppresses MAPK‐associated inflammatory and apoptotic pathways. (A) Gene Ontology (GO) enrichment analysis of DEGs in HDAC3‐CKO relative to HDAC3‐C. GO terms with *p*.adjust < .05 were considered significantly enriched. (B) Representative images of TUNEL staining (green) in mouse lung tissues, with corresponding fluorescence quantification. Blue indicates cell nuclei stained with DAPI (*n* = 6). Scale bars, 200 µm. (C) Representative images of TUNEL staining (green) in primary mouse AT2 cells, with corresponding fluorescence quantification. Blue indicates cell nuclei stained with DAPI (*n* = 6). Scale bars, 100 µm. (D) Representative images of TUNEL staining (green) in A549 cells, with corresponding fluorescence quantification. Blue indicates cell nuclei stained with DAPI (*n* = 6). Scale bars, 100 µm. (E) Representative Western blot and corresponding quantification of Bcl‐2 and Bax in mouse lung tissues (*n* = 6). (F) Representative Western blot and corresponding quantification of Bcl‐2 and Bax in primary mouse AT2 cells (*n* = 6). (G) Representative Western blot and corresponding quantification of Bcl‐2 and Bax in A549 cells (*n* = 6). (H) Heatmap of inflammation‐related genes selected from the DEGs. Most of these genes are significantly downregulated in HDAC3‐CKO relative to HDAC3‐C. (I) Protein–protein interaction (PPI) network of differentially expressed genes with a correlation threshold of 0.15. Nodes in red indicate genes upregulated in the HDAC3‐CKO relative to HDAC3‐C, whereas nodes in blue indicate genes downregulated in the HDAC3‐CKO relative to HDAC3‐C. (J) KEGG pathway enrichment analysis of DEGs in HDAC3‐CKO relative to HDAC3‐C. Pathways with *p*.adjust < .05 were considered significantly enriched. (K, L) Representative Western blot and corresponding quantification of MAPK pathway proteins (ERK, p‐ERK, JNK, p‐JNK, p38, p‐p38, c‐Fos) in primary mouse AT2 cells (*n* = 6). Differences were assessed by two‐way ANOVA. Values are presented as mean ± standard error of the mean (SEM). *p < .05, versus the indicated group; ns, not significant.

Based on these findings, we further characterised apoptosis. TUNEL staining showed a marked increase in apoptotic cells in the lung tissues of irradiated HDAC3‐C mice, whereas apoptosis was significantly reduced in HDAC3‐CKO mice (Figure 4B). In primary mouse AT2 cells and A549 cells, TUNEL staining similarly showed a significant increase in apoptotic cell numbers following irradiation (Figure 4C and D). Western blot analysis showed that in mouse lung tissues, primary mouse AT2 cells, and A549 cells, HDAC3 deficiency decreased Bax levels and increased Bcl‐2 levels, resulting in a significant reduction in the Bax/Bcl‐2 ratio (Figure 4E–G). Collectively, these results indicate that HDAC3 deficiency attenuates alveolar epithelial cell apoptosis in early‐stage RILI. Together with the RNA‐seq results indicating alterations in inflammatory signalling pathways, our results support a potential contribution of inflammatory responses and epithelial apoptosis to alveolar epithelial barrier dysfunction.

To further investigate the potential targets through which HDAC3 regulates apoptosis, we analysed differentially expressed inflammation‐related genes. Most pro‐inflammatory genes, including *CSF2*, *CXCL3*, and *CCL20*, showed significant downregulation in the HDAC3‐CKO group (Figure 4H), consistent with an attenuation of radiation‐induced inflammatory responses following HDAC3 inhibition. PPI network analysis identified *FOS* (c‐Fos) as a central node (Figure 4G), suggesting a potential role for c‐Fos in early‐stage RILI‐associated inflammatory signalling. c‐Fos belongs to the AP‐1 transcription factor family and can be directly activated by the MAPK signalling pathway or indirectly via the NF‐κB pathway. It forms a heterodimer with Jun to constitute the AP‐1 complex, thereby activating downstream inflammatory gene programmes.^37^ These findings led us to hypothesise that HDAC3 may regulate c‐Fos and thereby influence AP‐1 transcriptional activity, contributing to inflammatory responses and epithelial apoptotic processes in early‐stage RILI. To test this hypothesis, we first assessed AP‐1 transcriptional activity using a dual‐luciferase reporter assay. In A549 and MLE‐12 cells, irradiation increased AP‐1 transcriptional activity, whereas HDAC3 knockdown reduced IR‐induced AP‐1 transcriptional activity (Figure S5C), supporting a role for HDAC3 in regulating AP‐1 transcriptional activity in early‐stage RILI. In addition, knockdown of c‐Fos (knockdown efficiency validated in Figure S6A and B) attenuated radiation‐induced alveolar epithelial barrier damage and reduced apoptosis (Figure S6C and D), accompanied by decreased expression of inflammatory cytokines (Figure S6E and F). Combined overexpression of HDAC3 reversed these protective effects, further supporting a functional association between HDAC3 and c‐Fos in the regulation of early‐stage RILI‐associated inflammatory responses and epithelial apoptosis. Collectively, our results suggest that HDAC3 is involved in inflammation‐ and apoptosis‐related signalling through c‐Fos/AP‐1, which may influence alveolar epithelial barrier integrity.

Subsequently, KEGG analysis was conducted to characterise signalling pathways associated with HDAC3 deficiency. The differentially expressed genes showed significant enrichment in the MAPK pathway, indicating that MAPK‐related signalling may be involved in the transcriptional and pathological changes associated with HDAC3 deficiency (Figure 4J). Combined with the identification of *FOS* (c‐Fos) as a central node in the PPI network, these findings prompted us to further examine the major branches of the MAPK pathway to determine which MAPK signalling component was associated with HDAC3 deficiency. In primary mouse AT2 cells, we examined ERK1/2, JNK1/2/3, and p38 phosphorylation. Following irradiation stimulation, HDAC3 knockout did not significantly alter the phosphorylation levels of ERK1/2 or JNK1/2/3, whereas it reduced radiation‐induced p38 phosphorylation, accompanied by attenuated upregulation of c‐Fos protein expression (Figure 4K and L). These results indicate that, under HDAC3‐deficient conditions, the changes in MAPK signalling were primarily characterised by reduced activation of the p38 branch, accompanied by decreased c‐Fos expression, whereas no significant changes were observed in ERK1/2 or JNK1/2/3 phosphorylation. Previous studies have demonstrated that various external stressors activate p38‐MAPK, which subsequently regulates apoptotic and inflammatory processes.^38^ Taken together with the transcriptomic enrichment and protein‐level validation described above, these findings suggest that HDAC3 may regulate c‐Fos/AP‐1 activity via p38‐MAPK signalling, with potential consequences for inflammatory responses and epithelial apoptosis in early‐stage RILI and, consequently, alveolar epithelial barrier integrity.

### 3.5 HDAC3 regulates the p38/c‐Fos axis and radiation‐induced phenotypes via modulation of MKP1 acetylation

Mitogen‐activated protein kinase phosphatase 1 (MKP1) is a key negative regulator of the MAPK pathway, primarily dephosphorylating p38 MAPK and JNK to suppress the activation of downstream inflammatory mediators. Acetylation of MKP1 enhances its binding to p38 MAPK, thereby promoting p38 dephosphorylation and further inhibiting MAPK pathway activity.^39^ Previous studies have shown that inhibition of HDAC activity increases MKP1 acetylation, enhancing its suppression of MAPK signalling.^40^ These observations led us to hypothesise that, in RILI, HDAC3 may regulate MKP1 acetylation, thereby modulating its interaction with p38 MAPK and MAPK pathway activity, ultimately contributing to early‐stage RILI pathogenesis.

First, we assessed the effect of HDAC3 on MKP1 acetylation. Under irradiation, HDAC3 knockdown markedly increased MKP1 acetylation, whereas HDAC3 overexpression decreased it (Figure 5A). We next investigated how HDAC3 and MKP1 modulate p38 and the transcription factor c‐Fos. Western blot analysis revealed that neither HDAC3 nor MKP1 affected total p38 levels, but HDAC3 knockdown reduced phosphorylated p38 (p‐p38) and c‐Fos levels, and subsequent MKP1 knockdown reversed these changes (Figure 5B). Notably, previous studies have shown that inhibition of HDAC family members enhances MKP1 acetylation, promoting its binding to p38 MAPK and suppressing MAPK pathway activation.^41^ We therefore further evaluated MKP1‐p38 interaction in the RILI model. Results showed that under irradiation, HDAC3 knockdown enhanced MKP1 binding to p38, whereas HDAC3 overexpression reduced this interaction (Figure 5C). To further confirm these observations, we repeated the experiments in MLE‐12 cells. HDAC3 knockdown and overexpression produced similar effects on MKP1 acetylation, the MKP1–p38 interaction, and p38/c‐Fos signalling to those observed in A549 cells (Figure 5D–F). These findings suggest that HDAC3 may regulate c‐Fos expression by modulating MKP1 acetylation and thereby affecting the interaction between MKP1 and p38 and p38 phosphorylation.

**FIGURE 5:**
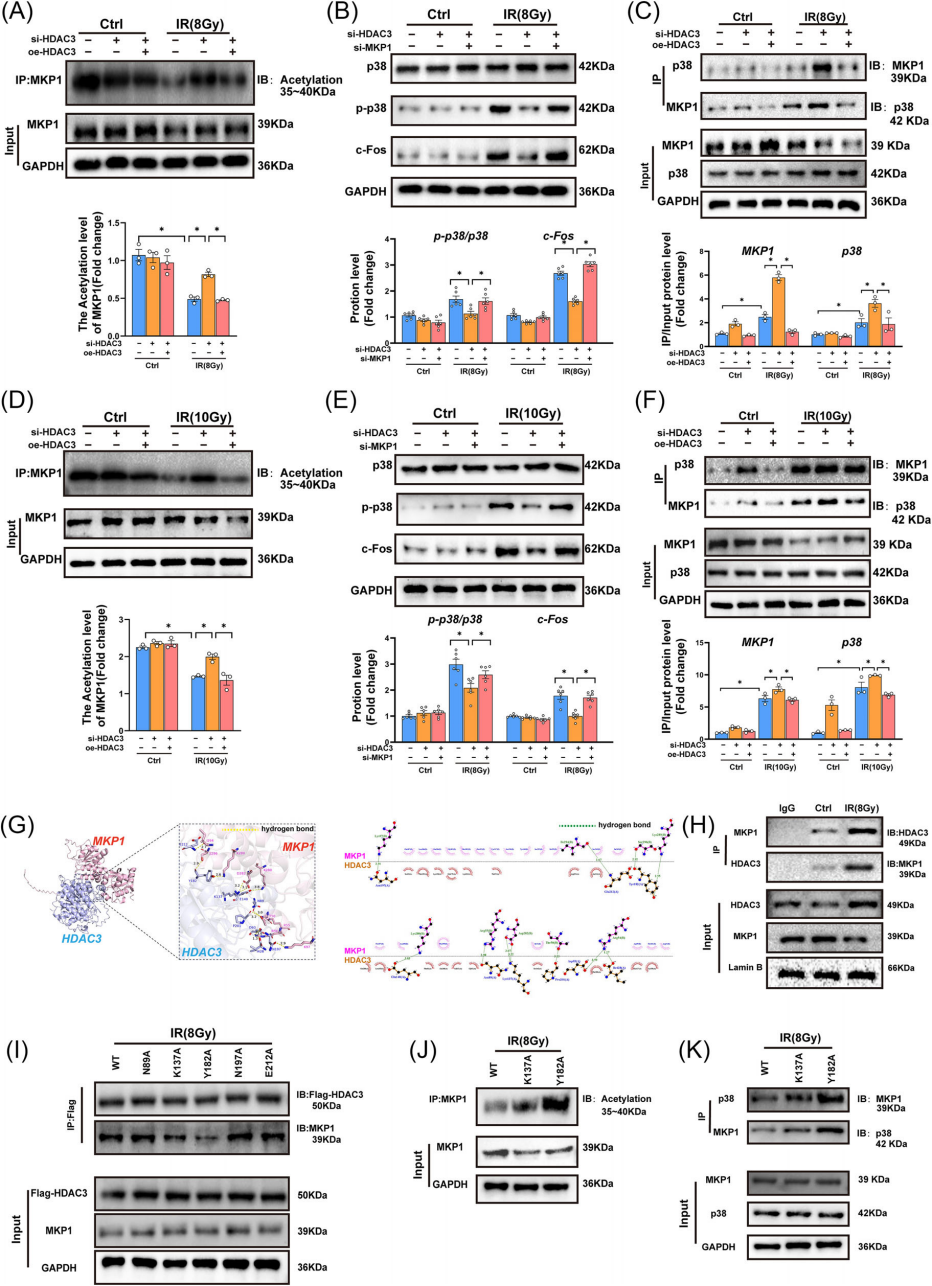
Molecular mechanism by which HDAC3 regulates MKP1 acetylation to modulate its interaction with p38. (A) Effect of HDAC3 on MKP1 acetylation levels in A549 cells. Representative Co‐IP analysis showing MKP1 immunoprecipitation and detection of acetylation levels, with corresponding quantification (*n* = 3). (B) Representative Western blot and corresponding quantification showing the effect of HDAC3 and MKP1 on P38, p‐p38, and c‐Fos in A549 cells (*n* = 6). (C) Representative Co‐IP analysis showing the effect of HDAC3 on MKP1‐p38 binding in A549 cells (*n* = 3). (D) Effect of HDAC3 on MKP1 acetylation levels in MLE‐12 cells. Representative Co‐IP analysis showing MKP1 immunoprecipitation and detection of acetylation levels, with corresponding quantification (*n* = 3). (E) Representative Western blot and corresponding quantification showing the effect of HDAC3 and MKP1 on P38, p‐p38, and c‐Fos in MLE‐12 cells (*n* = 6). (F) Representative Co‐IP analysis showing the effect of HDAC3 on MKP1‐p38 binding in MLE‐12 cells (*n* = 3). (G) Molecular docking analysis showing the binding site between HDAC3 and MKP1. (H) Representative Co‐IP analysis showing the binding between HDAC3 and MKP1 in the nucleus of A549 cells following irradiation (*n* = 3). Nuclear extracts were used for Co‐IP. (I) Immunoprecipitation with anti‐Flag beads in A549 cells following irradiation, transfected with Flag‐HDAC3 constructs containing mutations at the identified binding sites (*n* = 3). (J) Effect of HDAC3 K83 mutation on MKP1 acetylation levels in A549 cells following irradiation. Representative Co‐IP analysis showing MKP1 immunoprecipitation and detection of acetylation levels, with corresponding quantification (*n* = 3). (K) Representative Co‐IP analysis showing the effect of HDAC3 K83 mutation on MKP1‐p38 binding in A549 cells following irradiation (*n* = 3). Differences were assessed by two‐way ANOVA or Kruskal–Wallis test, as appropriate. Values are presented as mean ± standard error of the mean (SEM). *p < .05, versus the indicated group; ns, not significant.

To further define how MKP1 contributes to HDAC3‐mediated regulation of RILI, we performed combined knockdown experiments. The results showed that HDAC3 knockdown (knockdown efficiency validated in Figure S7A and B) substantially attenuated the RILI‐related phenotypic changes induced by MKP1 knockdown (Figure S7C–F), indicating a potential role for MKP1 in HDAC3‐mediated regulation of RILI‐related phenotypes. Subsequently, to further determine whether the regulation of MKP1 by HDAC3 depends on its deacetylase activity, we generated an HDAC3 Y298F mutant, which abolishes the deacetylase activity of HDAC3.^42^ Under irradiation, compared with wild‐type HDAC3, the HDAC3 Y298F mutant significantly increased MKP1 acetylation and enhanced the interaction between MKP1 and p38 (Figure S8A and B), suggesting involvement of HDAC3 deacetylase activity in regulating MKP1 acetylation and its interaction with p38. Collectively, our results are consistent with a potential involvement of the HDAC3–MKP1–p38–c‐Fos/AP‐1 signalling axis in RILI‐associated inflammation, apoptosis, and alveolar epithelial barrier injury.

To clarify the potential molecular mechanism by which HDAC3 regulates MKP1 acetylation, we first performed molecular docking analysis (Figure 5G). The results predicted that HDAC3 could directly interact with MKP1. After excluding implausible interactions based on bond lengths (including an unusually short distance of 1.27 Å and distances greater than 3.5 Å) and residue‐related considerations, five amino acid residues on HDAC3 were identified as potentially involved in the interaction with MKP1: Asn89, Lys137, Tyr182, Asn197, and Glu212 (Table S5). Considering that MKP1 is predominantly localised in the nucleus,^43^ we further performed nuclear‐cytoplasmic fractionation in A549 cells. The results showed that, under irradiation, HDAC3 predominantly interacted with nuclear MKP1, whereas relatively little interaction was observed in the cytoplasm (Figures 5H and S8C). In addition, irradiation stimulation similarly enhanced the interaction between HDAC3 and MKP1 in MLE‐12 cells (Figure S8D). These findings suggest that the interaction between HDAC3 and MKP1 occurs predominantly in the nucleus and may contribute to RILI‐associated signalling through regulation of nuclear MKP1 acetylation. To further verify whether HDAC3 and MKP1 undergo a direct protein‐protein interaction, we performed a GST pull‐down assay. We found that HDAC3 directly interacted with MKP1 (Figure S8E), further supporting a direct protein‐protein interaction between the two proteins. Subsequently, to further define the functional role of MKP1 acetylation site in this regulatory process, we generated the MKP1 K57R mutant.^40^ Under IR stimulation, the K57R mutation reduced MKP1 acetylation compared with MKP1 WT, whereas HDAC3 knockdown increased MKP1 acetylation levels (Figure S8F). Additionally, Co‐IP revealed that irradiation‐induced association between MKP1 and p38 was attenuated by the K57R mutation (Figure S8G). These findings indicate that K57 may participate in HDAC3‐mediated regulation of MKP1 acetylation. Furthermore, to validate the contribution of the predicted binding sites to the HDAC3–MKP1 interaction, we generated point mutants at the five predicted sites (N89A, K137A, Y182A, N197A, and E212A). Under irradiation, the K137A and Y182A mutations significantly reduced the interaction between HDAC3 and MKP1, whereas mutations at the other sites had relatively limited effects on their interaction (Figure 5I). Compared with the wild‐type protein, the K137A and Y182A mutations also increased MKP1 acetylation (Figure 5J) and enhanced the interaction between MKP1 and p38 (Figure 5K).

Collectively, our data indicate that HDAC3 regulates MKP1 acetylation and the subsequent MKP1–p38/c‐Fos signalling axis, which may contribute to RILI‐associated cellular phenotypes.

### 3.6 RGFP966‐mediated HDAC3 inhibition alleviates radiation‐induced alveolar epithelial injury and inflammatory responses

To explore the clinical potential of HDAC3 inhibition, we evaluated the protective effects of selective HDAC3 inhibition with RGFP966 (structure shown in Figure S9A; 10 mg/kg, i.p.) against radiation‐induced alveolar injury. RGFP966 is highly selective for HDAC3, with an IC_50_ of 80 nmol/L, and exhibits minimal inhibition of other HDACs even at concentrations up to 15 µmol/L.^24^

First, to assess drug safety, heart, liver, spleen, and kidney tissues were subjected to H&E staining. No obvious pathological changes were observed in RGFP966‐treated mice compared with the saline group (Figure S9B), indicating that the administered dose was well tolerated. Next, H&E staining of lung tissues from thorax‐irradiated mice revealed that RGFP966 markedly attenuated radiation‐induced pulmonary injury, including oedema, infiltration of inflammatory cells, and alveolar septal thickening (Figure 6A). Western blotting revealed that RGFP966 restored protein levels of the barrier‐related proteins ZO‐1, E‐cadherin, and Occludin, reduced Bax expression, while increasing Bcl‐2 expression (Figure 6B and C, RGFP966 concentration for cell treatment: 10 µM, dissolved in DMSO). Similar changes were observed in human primary alveolar type II epithelial cells (human primary AT2 cells) (Figure 6B and D, respectively). qPCR analysis further showed that RGFP966 decreased the transcript levels of these inflammatory mediators in both mouse lung tissues and human primary AT2 cells (Figure 6E). Further mechanistic validation in human primary AT2 cells showed that RGFP966 attenuated the radiation‐induced increase in p38 phosphorylation (Figure S9C). In addition, IR stimulation enhanced the interaction between HDAC3 and MKP1 (Figure S9D), whereas Co‐IP analysis showed that RGFP966 enhanced the interaction between MKP1 and p38 under irradiation (Figure S9E).

**FIGURE 6:**
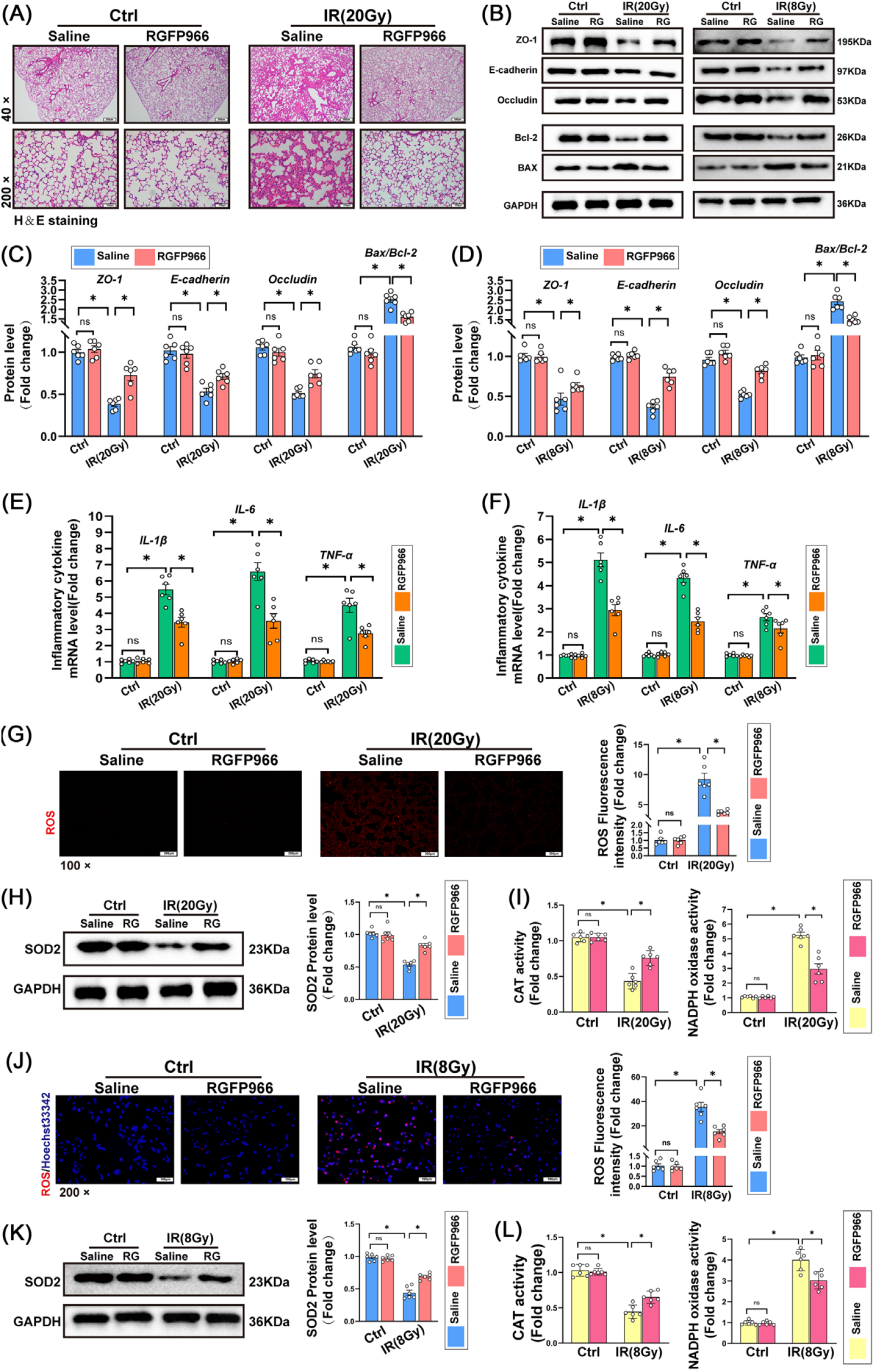
Pharmacological inhibition of HDAC3 by RGFP966 attenuates radiation‐induced alveolar barrier dysfunction, apoptosis, oxidative stress, and inflammation. (A) Representative H&E staining images of mouse lung tissues (*n* = 6). Scale bars, 500 and 100 µm, respectively. (B–D) Representative Western blot images and corresponding quantification of ZO‐1, E‐cadherin, Occludin, Bcl‐2, and Bax in mouse lung tissues and primary human AT2 cells (*n* = 6). (E) Relative mRNA levels of *IL‐1β*, *IL‐6*, and *TNF‐α* in mouse lung tissues (*n* = 6). (F) Relative mRNA levels of *IL‐1β*, *IL‐6*, and *TNF‐α* in primary human AT2 cells (*n* = 6). (G) Representative images of ROS fluorescence staining (red) in mouse lung tissues, with corresponding fluorescence quantification (*n* = 6). Scale bars, 200 µm. (H) Representative Western blot of SOD2 in mouse lung tissues (*n* = 6). (I) Markers of oxidative stress in mouse lung tissues, including catalase (CAT) and NADPH oxidase activities (*n* = 6). (J) Representative images of ROS fluorescence staining (red) in primary human AT2 cells, with corresponding fluorescence quantification. Blue indicates cell nuclei stained with Hoechst 33342 (*n* = 6). Scale bars, 100 µm. (K) Representative Western blot of SOD2 in primary human AT2 cells (*n* = 6). (L)Markers of oxidative stress in primary human AT2 cells, including catalase (CAT) and NADPH oxidase activities (*n* = 6).Differences were assessed by two‐way ANOVA. Values are presented as mean ± standard error of the mean (SEM). *p < .05, versus the indicated group; ns, not significant.

Notably, irradiation can induce ROS levels to rise to supra‐physiological levels, thereby causing oxidative damage to alveolar epithelial cells. This oxidative stress acts as a signalling cascade to sustain the release of pro‐inflammatory and pro‐fibrotic mediators, ultimately resulting in epithelial apoptosis, barrier dysfunction, and parenchymal fibrosis.^44^ Based on this mechanism, we next assessed changes in oxidative stress markers following RGFP966 treatment. ROS fluorescence staining showed that radiation significantly increased ROS levels in mouse lungs, whereas RGFP966 reduced ROS accumulation (Figure 6G). Western blot analysis confirmed that SOD2 expression was restored (Figure 6H). Further analysis of other oxidative stress‐related indicators showed that, compared with the saline group, RGFP966‐treated mice exhibited higher CAT activity and lower NADPH oxidase activity (Figure 6I). Similar changes were observed in human primary AT2 cells: ROS staining showed that irradiation significantly increased intracellular ROS levels, whereas Western blot analysis showed reduced SOD2 expression and dysregulated oxidative stress; RGFP966 effectively reversed these changes (Figure 6J–L).

In summary, RGFP966‐mediated HDAC3 inhibition attenuates radiation‐induced inflammation and apoptosis and preserves alveolar epithelial barrier integrity, as reflected by restored ZO‐1 and Occludin expression and modulation of Bax/Bcl‐2 levels. In addition, RGFP966 alleviates oxidative stress, as evidenced by increased SOD2 expression and CAT activity and reduced NADPH oxidase activity. Together, these findings support the protective effects of HDAC3 inhibition in early‐stage RILI and provide a basis for further investigation of HDAC3‐directed therapeutic approaches.

## 4 DISCUSSION

Our study showed that radiation exposure was associated with increased HDAC3 levels in AT2 cells and that HDAC3 may promote inflammatory responses and apoptosis through the MKP1–p38–c‐Fos/AP‐1 signalling axis, contributing to alveolar epithelial barrier dysfunction during early‐stage RILI. Genetic deletion of Hdac3 in AT2 cells and pharmacological inhibition of HDAC3 with RGFP966 attenuated radiation‐induced alveolar epithelial injury and reduced inflammation and apoptosis. Collectively, our results indicate that HDAC3 may regulate early‐stage RILI and support the HDAC3–MKP1–p38–c‐Fos/AP‐1 axis as a mechanism contributing to alveolar epithelial injury.

AT2 cells play important roles in maintaining alveolar epithelial barrier integrity and local microenvironmental homeostasis and are considered a key cell type in the development of RILI.^16^, ^17^, ^18^ Previous studies have demonstrated that HDAC3 participates in pulmonary inflammation, oxidative stress, and fibrosis and regulates alveolar epithelial cell apoptosis and barrier function.^22^, ^23^, ^24^, ^25^ However, the role of HDAC3 in RILI and its regulatory mechanisms in AT2 cells remain unclear. Our data showed that radiation exposure upregulated HDAC3 in AT2 cells, whereas AT2‐specific deletion of *HDAC3* attenuated radiation‐induced alveolar injury, consistent with the involvement of HDAC3 in AT2 cell responses to radiation injury. Notably, previous studies have shown that HDAC3 can exert distinct biological effects in different pulmonary disease contexts,^22^, ^23^, ^24^, ^25^ suggesting that its functions may depend on cell type and disease context. Our findings further link HDAC3 to specific signalling mechanisms in RILI and provide new insights into its involvement in radiation‐associated pulmonary injury.

The MAPK signalling pathway is an important regulatory network involved in inflammatory and apoptotic responses and comprises major members such as ERK, JNK, and p38 MAPK.^45^ MAPK activity is negatively regulated by MAPK phosphatases (MKPs), also referred to as dual‐specificity phosphatases (DUSPs), through dephosphorylation.^39^ Among them, MKP1 is a prototypical member of the MKP/DUSP family that preferentially dephosphorylates JNK and p38 MAPK^46^, ^47^ and participates in the regulation of inflammatory responses in various disease contexts.^43^, ^48^, ^49^ Consistent with previous studies, we found that MKP1 regulates inflammation, apoptosis, and barrier integrity in alveolar epithelial cells during early‐stage RILI. Notably, previous studies have identified a close relationship between MKP1 acetylation and its regulatory activity toward p38. Kim et al. reported that inhibition of HDAC activity increased MKP1 acetylation and promoted its binding to p38 in a sepsis model,^41^ while Cao et al. further showed that acetylation of MKP1 at Lys57 enhanced its dephosphorylation activity toward p38.^40^ Based on these findings, we further investigated whether HDAC3 regulates p38 signalling in early‐stage RILI through modulation of MKP1 acetylation. Our results showed that radiation exposure promoted the interaction between HDAC3 and MKP1, accompanied by reduced MKP1 acetylation and increased p38 phosphorylation. Conversely, HDAC3 inhibition increased MKP1 acetylation and enhanced its interaction with p38, together with reduced p38 phosphorylation. These findings support a role for HDAC3‐mediated MKP1 deacetylation in the regulation of p38 signalling during early‐stage RILI. Furthermore, AP‐1 is an important downstream transcriptional regulator of the MAPK signalling pathway and participates in various biological processes, including inflammation and apoptosis.^36^ We found that radiation exposure increased c‐Fos expression in alveolar epithelial cells, whereas HDAC3 deletion attenuated this response. Previous studies have shown that AP‐1 regulates the transcription of inflammation‐related mediators, including *IL‐1β*, *IL‐6*, *TNF‐α*, *CSF2*, *CXCL3*, and *CCL20*, and can influence epithelial barrier integrity through regulation of tight junction proteins.^50^ In addition, the BAX promoter contains two AP‐1 binding sites, indicating that AP‐1 may contribute to the transcriptional control of apoptosis‐related genes.^51^ Consistent with these findings, we found that reducing AP‐1 activity in alveolar epithelial cells attenuated radiation‐induced barrier disruption and reduced inflammation and apoptosis. Collectively, these findings support a potential role for the HDAC3–MKP1–p38–c‐Fos/AP‐1 axis in regulating inflammation, apoptosis, and alveolar epithelial barrier dysfunction during early‐stage RILI.

We next examined the effects of pharmacological HDAC3 inhibition on early‐stage RILI. Administration of the HDAC3‐specific inhibitor RGFP966 attenuated radiation‐induced alveolar epithelial barrier disruption, apoptosis, and inflammatory responses, consistent with the protective effects observed following AT2‐specific deletion of *HDAC3* and further supporting a role for HDAC3 in the pathogenesis of early‐stage RILI. RGFP966 exhibits high selectivity for HDAC3, with an IC_50_ of approximately 80 nM, and shows limited activity against other HDAC family members even at higher concentrations, supporting its use as a pharmacological tool for HDAC3 inhibition. Notably, previous studies have reported potential antitumour effects of HDAC3 inhibition in certain lung cancer models; for example, HDAC3 deficiency suppresses the growth of *Kras/LKB1*‐mutant lung tumours^52^ and can reverse osimertinib resistance caused by EGFR/BIM loss.^53^ Therefore, whether HDAC3‐targeted intervention can influence both lung tissue injury and tumour responses in the context of radiotherapy warrants further investigation. However, RGFP966 was not directly assessed for its effects on antitumour efficacy in a tumour radiotherapy model, and its potential effects in this context require further validation. In addition, the pharmacokinetics, safety, and long‐term effects of RGFP966 in early‐stage RILI intervention remain to be further evaluated.

It should be noted that oxidative stress is widely recognised as an important mechanism underlying IR‐induced tissue injury and is closely implicated in the pathogenesis of RILI. Prior evidence indicates that ROS accumulation contributes to alveolar epithelial injury, inflammatory responses, and subsequent tissue remodelling.^10^ However, transcriptomic analysis of HDAC3‐deficient primary mouse AT2 cells in the prior evidence revealed that the enriched pathways of differentially expressed genes were not primarily associated with oxidative stress‐related signalling, but were more closely related to inflammatory responses and MAPK‐related signalling networks, particularly the p38–c‐Fos axis. Therefore, our mechanistic investigations primarily focused on inflammation‐ and apoptosis‐related processes rather than oxidative stress as a core mechanism of HDAC3‐mediated RILI. Considering the well‐established involvement of oxidative stress in radiation biology, our analysis next focused on oxidative stress‐related phenotypes. HDAC3 intervention was accompanied by improvements in oxidative stress‐related indicators; however, there is currently no direct evidence that these changes represent a downstream effect of the HDAC3–MKP1–p38–c‐Fos/AP‐1 signalling axis. Thus, the current findings more strongly support inflammation‐ and apoptosis‐related signalling as the primary mechanisms underlying HDAC3‐mediated early‐stage RILI, whereas the improvement in oxidative stress may represent an associated or secondary effect. Nevertheless, we cannot exclude potential interactions between oxidative stress, inflammation, and apoptosis. Additional investigation is warranted to define how oxidative stress contributes to HDAC3‐mediated RILI and relates to the signalling axis described above. At the same time, we cannot exclude the possibility that HDAC3 plays distinct roles in other pulmonary cell types during RILI. For example, our previous study showed that HDAC3 exacerbated sepsis‐associated lung injury by promoting macrophage pyroptosis,^27^ suggesting that the effects of HDAC3 may be cell type‐dependent. Future studies using conditional knockout models targeting different pulmonary cell types may further clarify the role of HDAC3 in different cellular compartments during early‐stage RILI.

From the perspective of precision medicine and clinical translation, the HDAC3–MKP1–p38–c‐Fos/AP‐1 axis identified in this study may provide a potential direction for further exploring patient stratification and individualised intervention in RILI. In recent years, multi‐omics integration, artificial intelligence, and large‐scale biomedical database analysis have increasingly been applied to molecular subtype identification, biomarker discovery, and treatment response prediction, providing new approaches for precision patient stratification.^54^, ^55^ Future studies could integrate genomic, transcriptomic, epigenomic, and clinical phenotypic data with machine learning, drug sensitivity prediction, and drug repurposing approaches to identify molecular features associated with HDAC3–MKP1–p38–c‐Fos/AP‐1 axis activity and RILI susceptibility and to explore HDAC3‐targeted interventions for different patient subgroups.^56^ Ultimately, integrating clinical characteristics, pulmonary imaging phenotypes, and molecular biomarkers, followed by validation in independent clinical cohorts, may facilitate the development of predictive models for RILI risk and response to HDAC3‐targeted interventions, thereby promoting the translation of these mechanistic findings toward precision treatment.^57^

In summary, this study further clarifies the role of HDAC3 in early‐stage RILI and its potential molecular regulatory mechanisms. Our findings suggest that HDAC3 may influence p38–c‐Fos/AP‐1 signalling by regulating MKP1 acetylation and thereby contribute to inflammatory responses, apoptosis, and alveolar epithelial barrier dysfunction. HDAC3 deficiency, achieved through either genetic deletion or pharmacological inhibition, attenuated these pathological changes, suggesting that HDAC3 may represent a therapeutic target for early‐stage RILI. Several limitations should be noted. First, primary AT2 cells are relatively fragile and difficult to genetically manipulate; therefore, A549 and MLE‐12 cell lines were used for some mechanistic studies. As neither cell line fully recapitulates the biological characteristics of normal primary AT2 cells, the mechanistic findings obtained from these models should be extrapolated to normal AT2 cells with caution. Although we further validated key findings in primary human AT2 cells, biological differences among different cell models cannot be completely excluded. Second, although our study further identified HDAC3 Lys137 and Tyr182 as residues associated with its interaction with MKP1, the complete protein–protein interaction interface between HDAC3 and MKP1, as well as other potential interaction sites, remains to be further characterised. In addition, other potential MKP1 acetylation sites and their functional roles warrant further investigation. Although analysis of a public human alveolar transcriptomic dataset provided additional evidence for the relevance of HDAC3 to radiation‐induced lung injury, direct validation in clinical specimens was not performed. Future studies using well‐characterised patient cohorts are warranted to further assess the clinical relevance of the HDAC3–MKP1–p38/c‐Fos axis. Finally, our investigation was centred on early‐stage RILI, whereas the involvement of HDAC3 in subsequent fibrotic remodelling and the transition from RILI to RIPF remains to be determined. Collectively, our results support the involvement of HDAC3 in early‐stage RILI and its associated signalling regulation and suggest that further preclinical investigation of HDAC3‐targeted interventions is warranted.

## 5 CONCLUSIONS

In summary, radiation induces inflammatory responses and apoptosis in AT2 cells, contributing to disruption of alveolar epithelial barrier integrity. Under conditions of HDAC3 upregulation, HDAC3 interacts with MKP1 and promotes its deacetylation, thereby enhancing p38 MAPK phosphorylation and c‐Fos expression and increasing AP‐1 transcriptional activity. This signalling axis is associated with increased expression of downstream pro‐inflammatory cytokines and may contribute to the development of early‐stage RILI (Figure 7). Our findings support a regulatory role for the HDAC3–MKP1–p38–c‐Fos/AP‐1 axis in RILI‐associated inflammation, apoptosis, and alveolar epithelial barrier dysfunction. Pharmacological inhibition of HDAC3 with RGFP966 attenuated these pathological changes, further supporting the potential of HDAC3 as a therapeutic target for limiting radiation‐induced lung injury. In addition, the improvement in oxidative stress‐related phenotypes following HDAC3 inhibition may be associated, at least in part, with reduced inflammatory and apoptotic responses, providing a basis for further investigation of HDAC3‐targeted strategies in early‐stage RILI.

**FIGURE 7:**
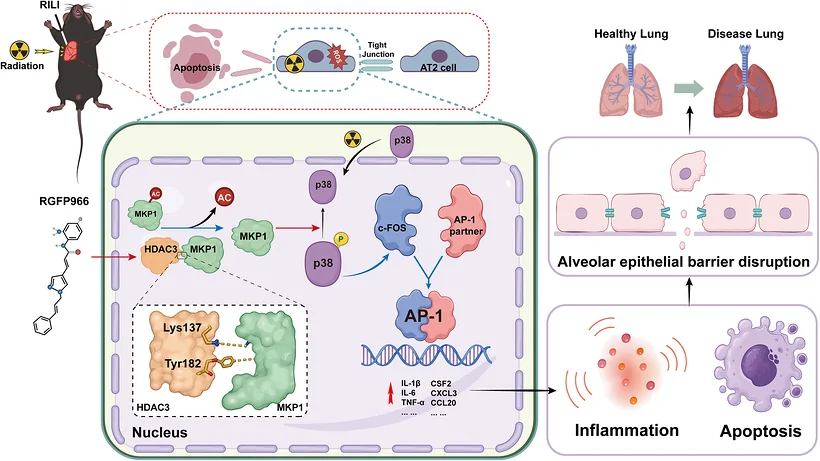
Proposed model of the HDAC3–MKP1–p38/c‐Fos axis driving RILI. Radiation induces ROS generation by ionising water molecules, which promotes AT2 cell apoptosis and damages alveolar barrier integrity. HDAC3 interacts with MKP1 at their respective binding sites (Lys137 and Tyr182 in HDAC3), deacetylating MKP1 to increase p38 phosphorylation, activating c‐Fos transcription, and enhancing AP‐1 activity. This cascade promotes inflammatory responses and apoptosis, which ultimately contribute to alveolar barrier disruption and the progression of RILI. The pharmacological inhibition of HDAC3 using RGFP966 may have therapeutic potential for RILI.Some elements of the schematic were created with BioGDP.com.^58^

## AUTHOR CONTRIBUTIONS

Boyang Li, Ning Li, and Rui Xiong contributed to the conception of the study, designed experiments, and were responsible for the overall work. Boyang Li, Yifan Zuo, Yi Liu, Guoqing Luo, and Wenjun Zhang performed experiments and conducted formal analysis. Min Peng, Long Wu, and Zhuya Xiao provided resources, supervised the project, and acquired funding. All authors read and approved the final manuscript.

## CONFLICT OF INTEREST STATEMENT

The authors declare no conflicts of interest.

## ETHICS STATEMENT

Animal experiments were approved by the Animal Ethics Committee of Renmin Hospital, Wuhan University (Approval Number: WDRM20250007) and were performed in strict accordance with the Guide for the Care and Use of Laboratory Animals published by the US National Institutes of Health. This study did not involve human participants.

## Supporting information



Supporting Information

## Data Availability

All data that support the findings in this study are available from the corresponding author upon reasonable request. The RNA‐seq data generated and analysed in this study have been deposited in the Genome Sequence Archive (GSA) at the National Genomics Data Center (NGDC), under the accession number CRA037842, and are publicly available.
