# Supplementary material for: HDAC3 drives type II alveolar epithelial barrier dysfunction via MKP1 deacetylation in radiation‐induced lung injury
Source: Clin Transl Med. 2026 Nov 9;16(11):e70877. doi: 10.1002/ctm2.70877 (PMC13746322; doi:10.1002/ctm2.70877)
Supplement: Supplementary file 1 [file CTM2-16-e70877-s001.pdf]

Supplementary figure 1. Expression of *HDAC1* and *HDAC2* in lung tissues following irradiation.

(A) *HDAC1* and *HDAC2* expression in lung tissues from rhesus macaques exposed to 9–12 Gy thoracic irradiation or control treatment (GSE70943). **(B)** *HDAC1* and *HDAC2* expression in lung tissues from mice exposed to 17.5 Gy thoracic irradiation or control treatment (GSE41789).

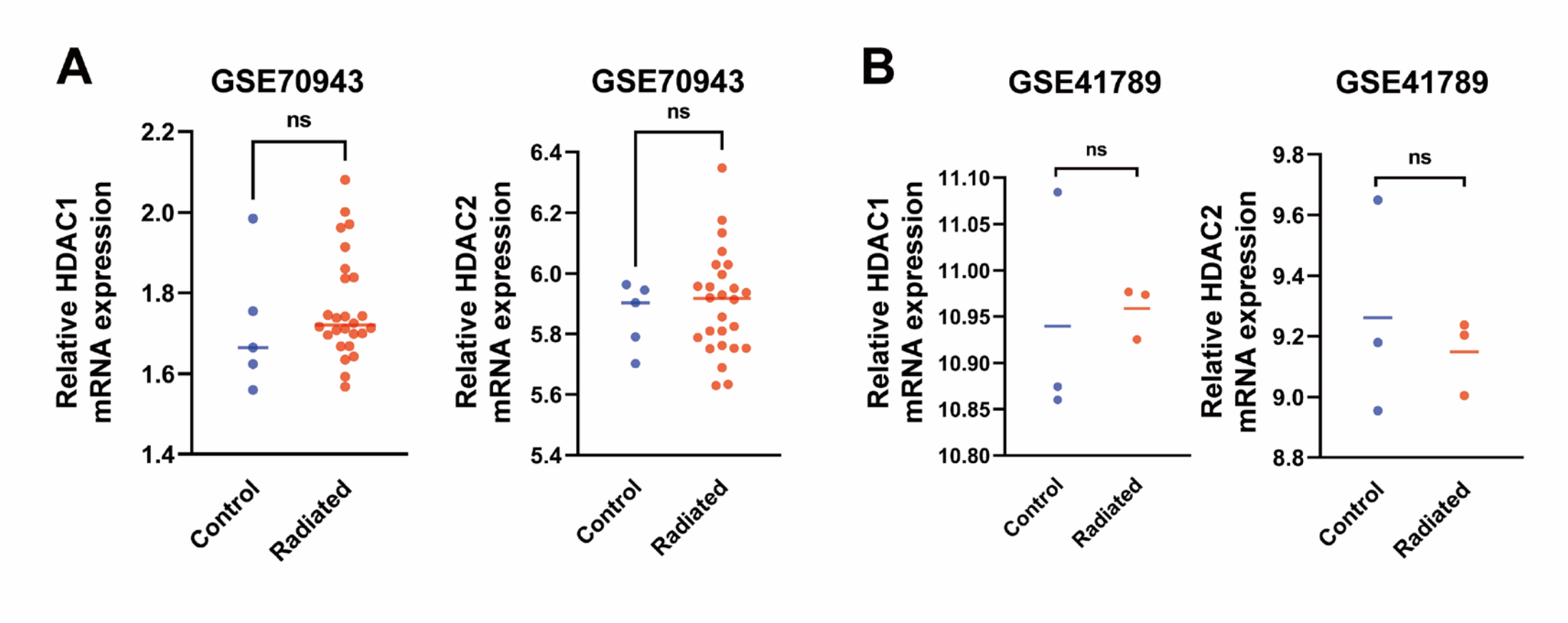

Supplementary figure 2. Radiation induces *HDAC3* and pro-inflammatory cytokine expression at the mRNA level.

(A) Relative mRNA levels of *HDAC3*, *IL-1β*, *IL-6*, and *TNF-α* in murine lung tissues following thoracic irradiation or control treatment (n = 6). **(B)** Relative mRNA levels of *HDAC3*, *IL-1β*, *IL-6*, and *TNF-α* in primary AT2 cells isolated from mice, following in vitro irradiation or control treatment (n = 6). **(C)** Relative mRNA levels of *HDAC3*, *IL-1β*, *IL-6*, and *TNF-α* in A549 cells following in vitro irradiation or control treatment (n = 6). **(D)** Relative mRNA levels of *HDAC3*, *IL-1β*, *IL-6*, and *TNF-α* in MLE-12 cells following in vitro irradiation or control treatment (n = 6). Differences were assessed by Student's *t* test or one-way ANOVA. Values are presented as mean ± standard error of the mean (SEM). $*p < 0.05$, versus the indicated group; ns, not significant.

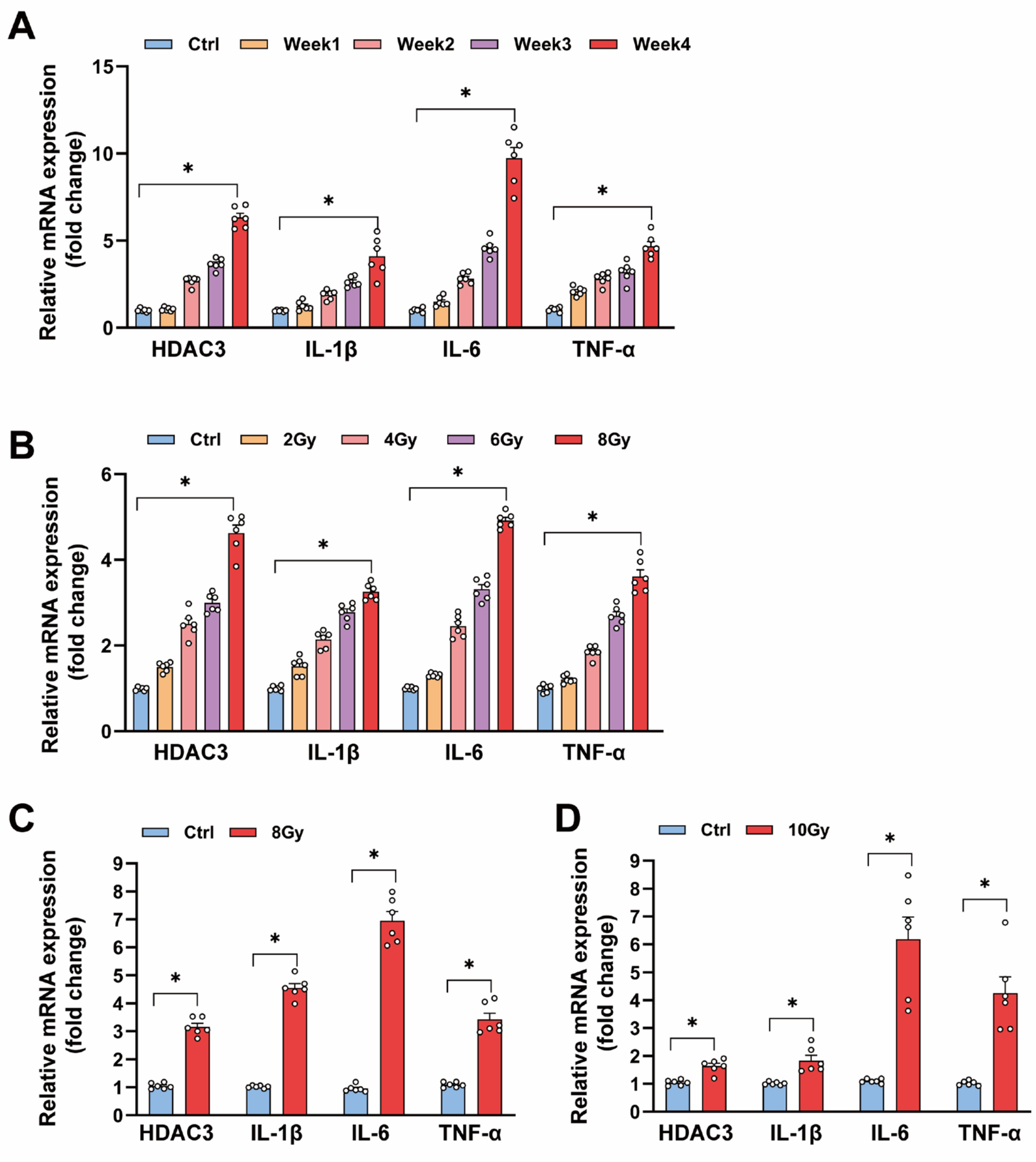

Supplementary figure 3. HDAC3 conditional knockout and knock-down validation.

(A) PCR-based genotyping of conditional alleles via 1.5% agarose gel electrophoresis. **(i)** HDAC3-flox locus (top) : wild-type mice generate a single 290 bp amplicon; heterozygous generate both 290 bp and 356 bp amplicons; homozygous mice generate a single 356 bp amplicon. **(ii)** *Sftpc*-Cre transgene (bottom) : wild-type mice generate the WT band; heterozygous mice generate both WT and 3′arm bands; homozygous mice generate only the 3′arm band. **(B)** Relative mRNA level of *HDAC3* in murine lung tissues from HDAC3-C or HDAC3-CKO mice (n = 6). **(C)** Relative mRNA level of *HDAC3* in primary AT2 cells isolated from HDAC3-C or HDAC3-CKO mice (n = 3). **(D)** Relative mRNA level of *HDAC3* in A549 cells transfected with non-targeting control siRNA (si-NTC, hereafter si-NC) or si-HDAC3 (n = 3). **(E)** Relative mRNA level of *HDAC3* in MLE-12 cells transfected with non-targeting control siRNA (si-NTC, hereafter si-NC) or si-HDAC3 (n = 3). Differences were assessed by Student's *t* test.Values are presented as mean ± standard error of the mean (SEM). *$p < 0.05$, versus the indicated group; ns, not significant.

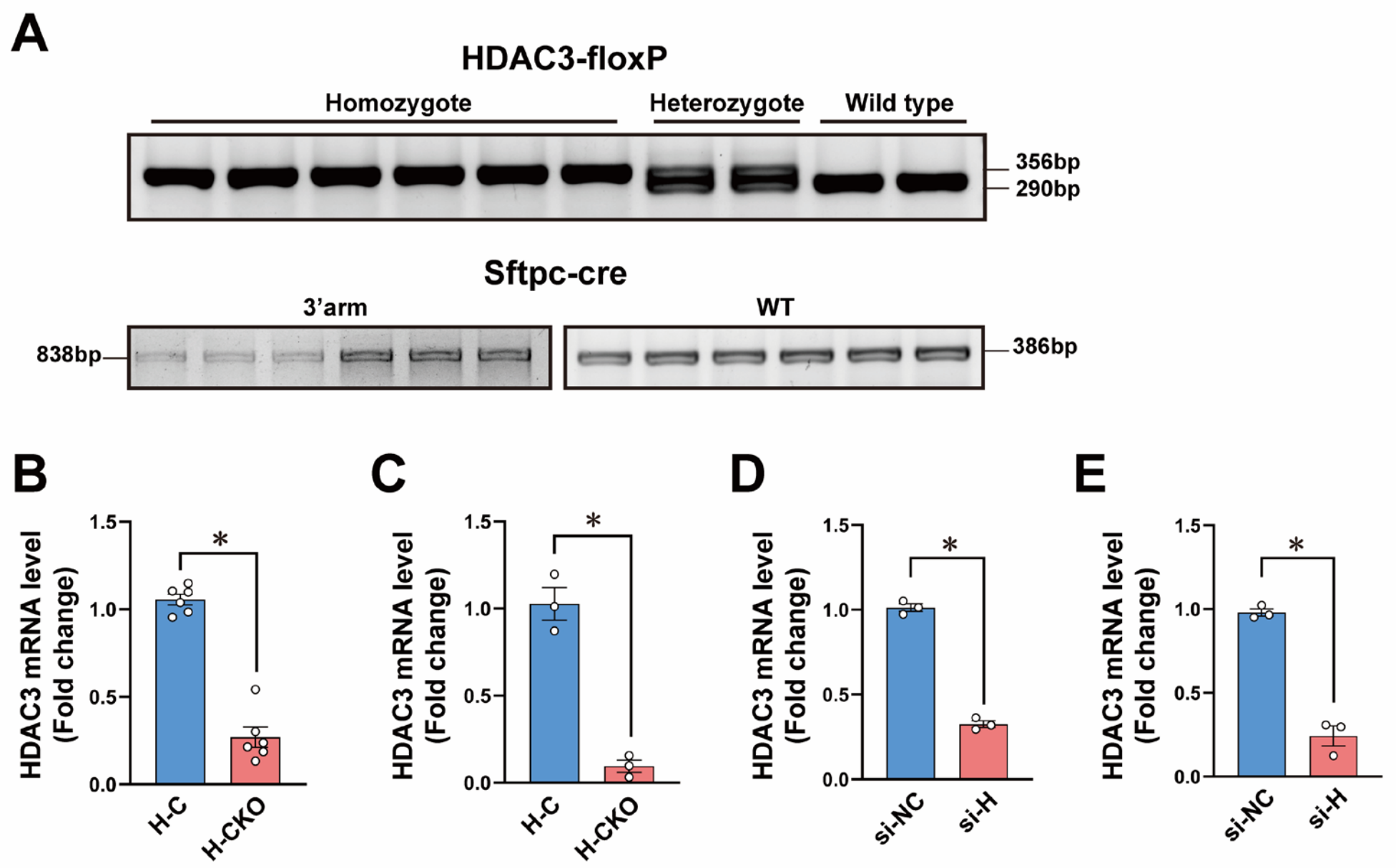

Supplementary figure 4. Individual fluorescence channels of ZO-1, Occludin, and E-cadherin staining in mouse lung tissues

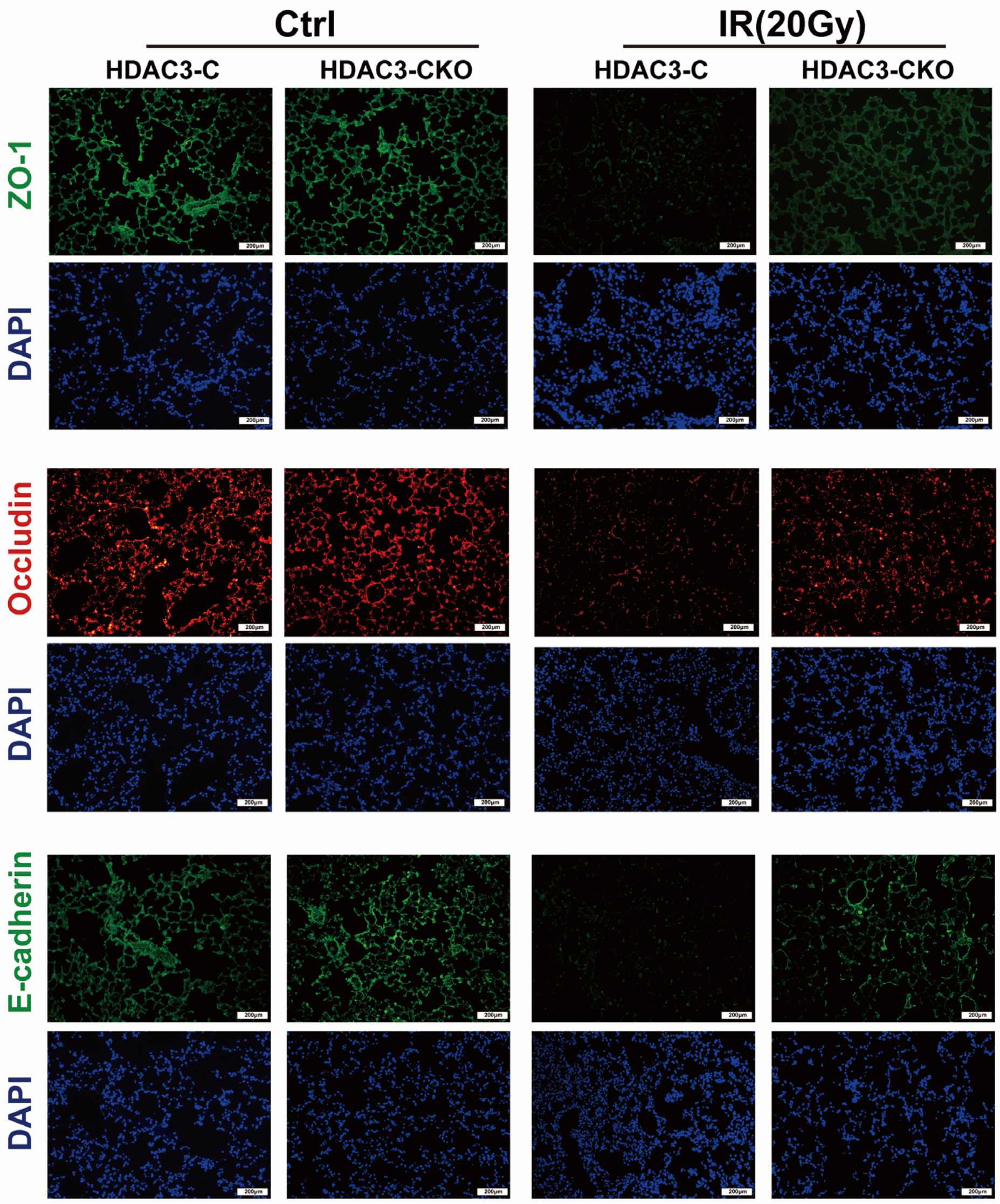

Supplementary figure 5. Differential gene expression analysis in irradiated primary AT2 cells from HDAC3-CKO versus HDAC3-C mice.
(A) Volcano plot showing differentially expressed genes (DEGs) in primary AT2 cells from HDAC3-CKO relative to HDAC3-C. DEGs were identified using edgeR with a FDR < 0.05 and | fold change |≥1.2. Red dots indicate upregulated genes, blue dots indicate downregulated genes, and gray dots represent genes without significant change. **(B)** Heatmap of all differentially expressed genes (DEGs) in HDAC3-CKO relative to HDAC3-C primary AT2 cells. Lines indicate genes related to inflammation. **(C)** Schematic illustration of the AP-1 luciferase reporter plasmid and AP-1 transcriptional activity in A549 and MLE-12 cells following irradiation, as determined by an AP-1 luciferase reporter assay. Differences were assessed by two-way ANOVA. Values are presented as mean ± standard error of the mean (SEM). *$p < 0.05$, versus the indicated group; ns, not significant.

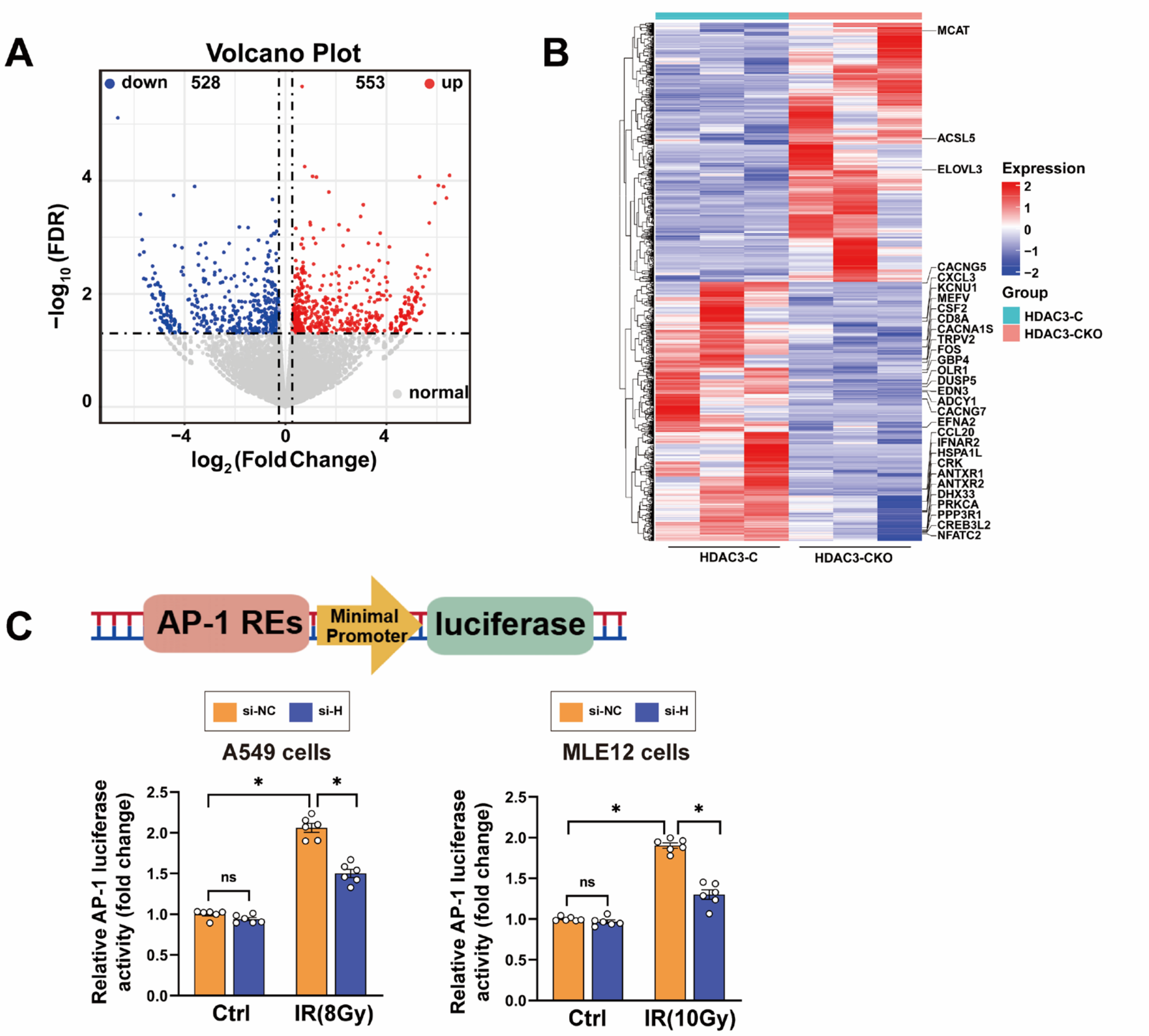

Supplementary figure 6. Combined FOS knockdown and HDAC3 overexpression reveal their effects on alveolar barrier integrity, radiation-induced apoptosis, and inflammation in RILI.

(A) Western blot and corresponding quantification of c-Fos in A549 cells transfected with si-NC and si-FOS (n = 3). **(B)** Western blot and corresponding quantification of c-Fos in MLE-12 cells transfected with si-NC and si-FOS (n = 3). **(C)** Representative Western blot and corresponding quantification of ZO-1, E-cadherin, Occludin, BAX and Bcl-2 in A549 cells (n = 6). **(D)** Representative Western blot and corresponding quantification of ZO-1, E-cadherin, Occludin, BAX and Bcl-2 in MLE-12 cells (n = 6). **(E)** Relative mRNA levels of *IL-1β*, *IL-6*, and *TNF-α* in A549 cells (n = 6). **(F)** Relative mRNA levels of *IL-1β*, *IL-6*, and *TNF-α* in MLE-12 cells (n = 6). Differences were assessed by two-way ANOVA.Values are presented as mean ± standard error of the mean (SEM). *$p < 0.05$, versus the indicated group; ns, not significant.

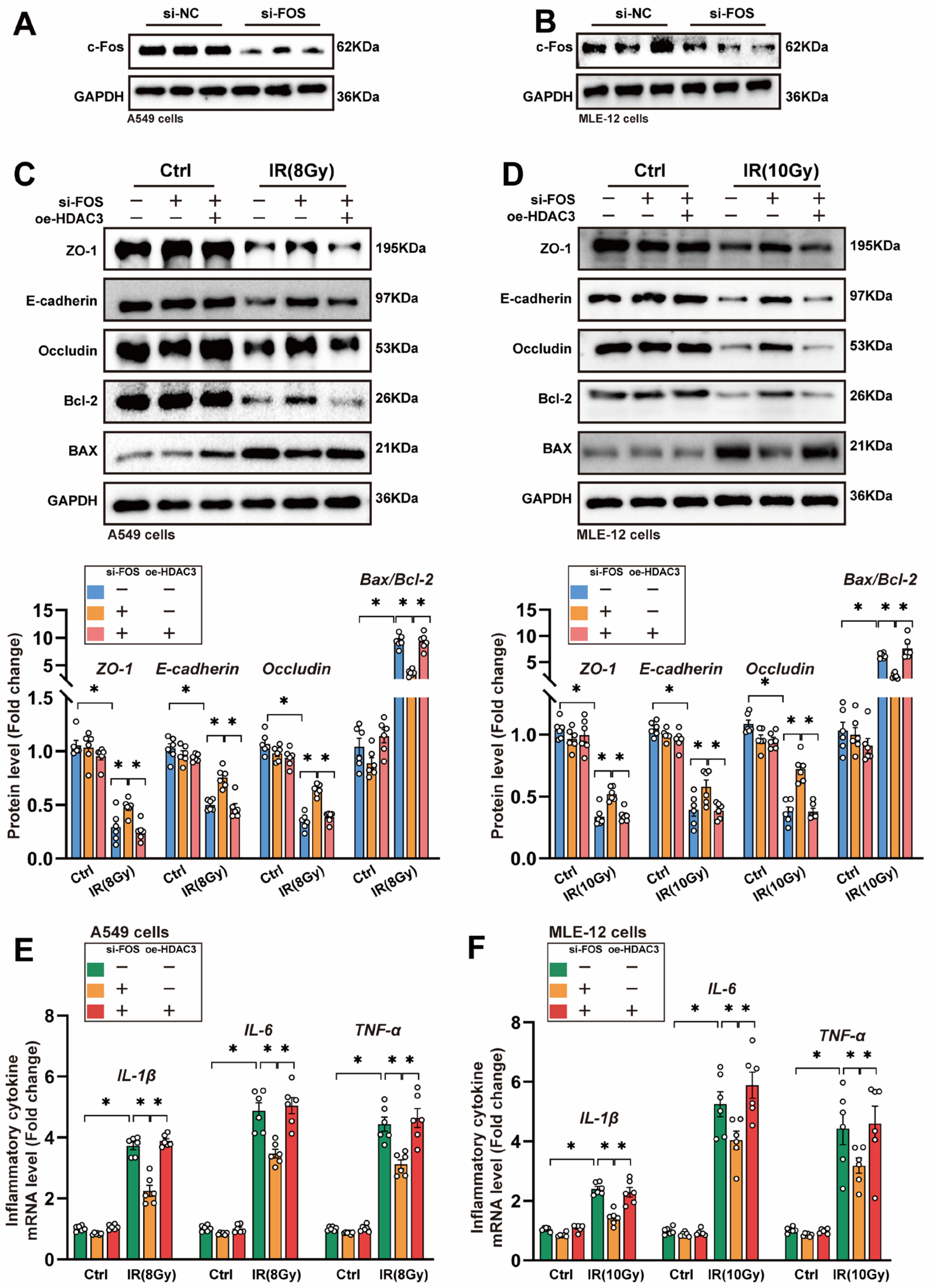
A
si-NC
si-FOS
c-Fos
62KDa
GAPDH
36KDa
A549 cells
B
si-NC
si-FOS
c-Fos
62KDa
GAPDH
36KDa
MLE-12 cells
C
Ctrl
IR(8Gy)
si-FOS
oe-HDAC3
ZO-1
195KDa
E-cadherin
97KDa
Occludin
53KDa
Bcl-2
26KDa
BAX
21KDa
GAPDH
36KDa
A549 cells
D
Ctrl
IR(10Gy)
si-FOS
oe-HDAC3
ZO-1
195KDa
E-cadherin
97KDa
Occludin
53KDa
Bcl-2
26KDa
BAX
21KDa
GAPDH
36KDa
MLE-12 cells
Protein level (Fold change)
ZO-1
E-cadherin
Occludin
Bax/Bcl-2
Ctrl
IR(8Gy)
IR(10Gy)
E
A549 cells
Inflammatory cytokine mRNA level (Fold change)
IL-1β
IL-6
TNF-α
F
MLE-12 cells

Supplementary figure 7. Combined MKP1 knockdown and HDAC3 knockdown reveal their effects on alveolar barrier integrity, radiation-induced apoptosis, and inflammation in RILI.

(A) Western blot and corresponding quantification of MKP1 in A549 cells transfected with si-NC and si-MKP1 (n = 3). **(B)** Western blot and corresponding quantification of MKP1 in MLE-12 cells transfected with si-NC and si-MKP1 (n = 3). **(C)** Representative Western blot and corresponding quantification of ZO-1, E-cadherin, Occludin, BAX and Bcl-2 in A549 cells (n = 6). **(D)** Representative Western blot and corresponding quantification of ZO-1, E-cadherin, Occludin, BAX and Bcl-2 in MLE-12 cells (n = 6). **(E)** Relative mRNA levels of *IL-1β*, *IL-6*, and *TNF-α* in A549 cells (n = 6). **(F)** Relative mRNA levels of *IL-1β*, *IL-6*, and *TNF-α* in MLE-12 cells (n = 6). Differences were assessed by two-way ANOVA.Values are presented as mean ± standard error of the mean (SEM). *$p < 0.05$, versus the indicated group; ns, not significant.

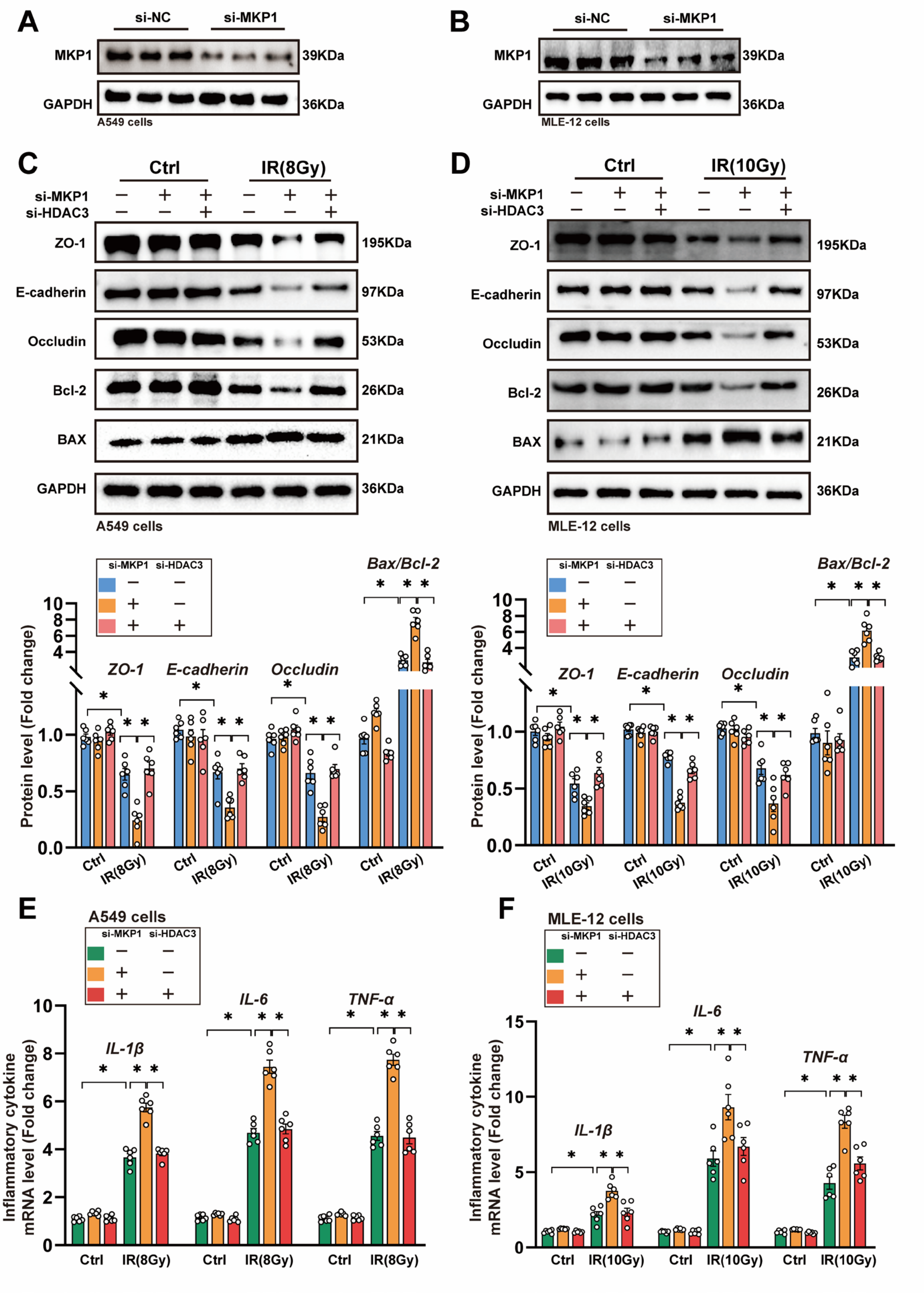
A
si-NC
si-MKP1
MKP1
39KDa
GAPDH
36KDa
A549 cells
B
si-NC
si-MKP1
MKP1
39KDa
GAPDH
36KDa
MLE-12 cells
C
Ctrl
IR(8Gy)
si-MKP1
si-HDAC3
ZO-1
195KDa
E-cadherin
97KDa
Occludin
53KDa
Bcl-2
26KDa
BAX
21KDa
GAPDH
36KDa
A549 cells
D
Ctrl
IR(10Gy)
si-MKP1
si-HDAC3
ZO-1
195KDa
E-cadherin
97KDa
Occludin
53KDa
Bcl-2
26KDa
BAX
21KDa
GAPDH
36KDa
MLE-12 cells
Protein level (Fold change)
ZO-1
E-cadherin
Occludin
Bax/Bcl-2
Ctrl
IR(8Gy)
IR(10Gy)
E
A549 cells
si-MKP1 si-HDAC3
Inflammatory cytokine mRNA level (Fold change)
IL-1β
IL-6
TNF-α
Ctrl
IR(8Gy)
F
MLE-12 cells
si-MKP1 si-HDAC3
Inflammatory cytokine mRNA level (Fold change)
IL-1β
IL-6
TNF-α
Ctrl
IR(10Gy)

Supplementary figure 8. Validation of HDAC3–MKP1 interaction and the role of MKP1 K57 in HDAC3-mediated regulation of MKP1 acetylation and MKP1–p38 interaction. (A) Effect of HDAC3 Y298 mutation on MKP1 acetylation levels in A549 cells following irradiation. Representative Co-IP analysis showing MKP1 immunoprecipitation and detection of acetylation levels, with corresponding quantification (n = 3). **(B)** Representative Co-IP analysis showing the effect of HDAC3 Y298 mutation on MKP1-p38 binding in A549 cells following irradiation (n = 3). **(C)** Representative Co-IP analysis showing the lack of binding between HDAC3 and MKP1 in the cytoplasm of A549 cells following irradiation (n = 3). Cytoplasmic extracts were used for Co-IP. **(D)** Representative Co-IP analysis showing the interaction between HDAC3 and MKP1 in MLE-12 cells following irradiation (n = 3). **(E)** GST pull-down assay showing the direct interaction between GST-tagged HDAC3 and His-tagged MKP1. **(F)** Effect of HDAC3 knockdown and MKP1 K57R mutation on MKP1 acetylation levels in A549 cells following irradiation. Representative Co-IP analysis showing MKP1 immunoprecipitation and detection of its acetylation levels, with corresponding quantification (n = 3). **(G)** Representative Co-IP analysis showing the effect of the MKP1 K57R mutation on MKP1–p38 interaction in A549 cells following irradiation (n = 3). Differences were assessed by the Kruskal–Wallis test. Values are presented as mean ± standard error of the mean (SEM). *$p < 0.05$, versus the indicated group; ns, not significant.

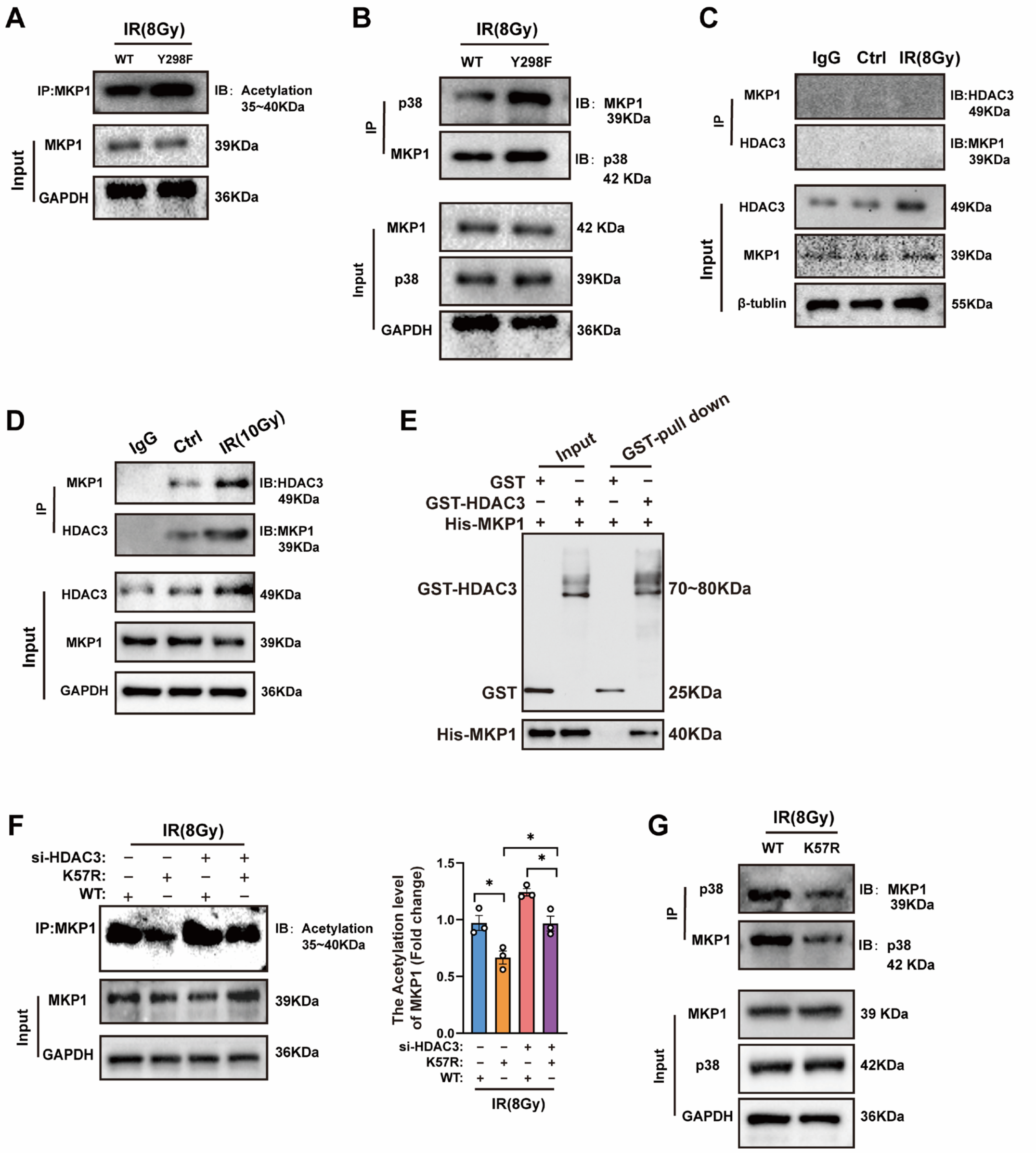
A
IR(8Gy)
WT
Y298F
IP:MKP1
IB: Acetylation 35~40KDa
Input
MKP1
39KDa
GAPDH
36KDa
B
IR(8Gy)
WT
Y298F
IP
p38
IB: MKP1 39KDa
MKP1
IB: p38 42 KDa
Input
MKP1
42 KDa
p38
39KDa
GAPDH
36KDa
C
IgG
Ctrl
IR(8Gy)
IP
MKP1
IB:HDAC3 49KDa
HDAC3
IB:MKP1 39KDa
Input
HDAC3
49KDa
MKP1
39KDa
β-tublin
55KDa
D
IgG
Ctrl
IR(10Gy)
IP
MKP1
IB:HDAC3 49KDa
HDAC3
IB:MKP1 39KDa
Input
HDAC3
49KDa
MKP1
39KDa
GAPDH
36KDa
E
Input
GST-pull down
GST
GST-HDAC3
His-MKP1
GST-HDAC3
70~80KDa
GST
25KDa
His-MKP1
40KDa
F
IR(8Gy)
si-HDAC3:
K57R:
WT:
IP:MKP1
IB: Acetylation 35~40KDa
Input
MKP1
39KDa
GAPDH
36KDa
The Acetylation level of MKP1 (Fold change)
1.5
1.0
0.5
0.0
*
si-HDAC3:
K57R:
WT:
IR(8Gy)
G
IR(8Gy)
WT
K57R
IP
p38
IB: MKP1 39KDa
MKP1
IB: p38 42 KDa
Input
MKP1
39 KDa
p38
42KDa
GAPDH
36KDa

Supplementary figure 9. Additional validation of RGFP966 effects in mice and primary human AT2 cells. (A) The molecular structure of RGFP966. The structure was created with BioGDP.com [58]. **(B)** 6–8 week-old mice were treated with Saline or RGFP966 and sacrificed for histopathological examination. Representative H&E-stained sections of the heart, liver, spleen, lung, and kidney showed no evident pathological alterations. Scale bars are shown in the images. **(C)** Representative Western blot images of p38 and p-p38 in primary human AT2 cells (n = 6). **(D)** Representative Co-IP analysis showing the interaction between HDAC3 and MKP1 in primary human AT2 cells following irradiation (n = 3). **(E)** Representative Co-IP analysis showing the effect of RGFP966 on MKP1-p38 binding in primary human AT2 cells following irradiation (n = 3).

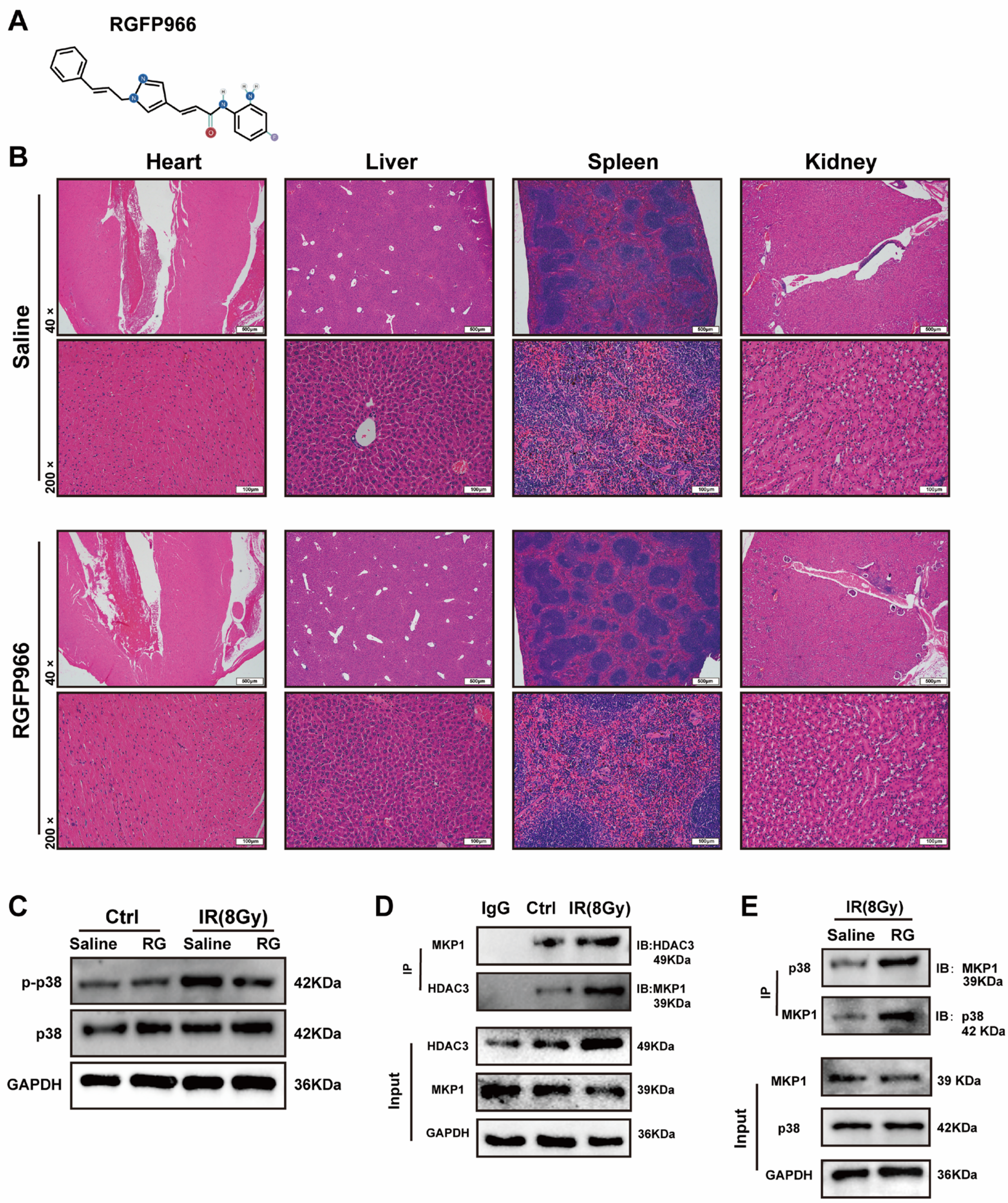

Supplementary Table S1. Primary antibodies used in the present study

Antibody	Customer	Product code	Dilution	Application
HDAC3	Abcam	ab32369	1:50~1:5000	WB, IF, CO-IP
GAPDH	Abcam	ab8245	1:10000	WB,
β-tublin	Abcam	ab179511	1:1000	WB
Lamin-B	Abcam	ab229025	1:1000	WB
BAX	Abcam	ab32503	1:5000	WB
BCL-2	Abcam	ab32124	1:1000	WB
ZO-1	ABclonal	A0650	1:50~1000	WB, IF
E-cad	ABclonal	A20798	1:2000	WB
Occludin	ABclonal	A2601	1:50~1000	WB, IF
SOD2	ABclonal	A21805	1:1000	WB
ERK	Bioworld	BS1968	1:1000	WB
p-ERK	Bioworld	AP0490	1:1000	WB
JNK	Bioworld	BS3630	1:1000	WB
p-JNK	Bioworld	BS4763	1:1000	WB
p38	Abcam	ab170099	1:50~5000	WB, CO-IP
p-p38	Bioworld	BS6381	1:2000	WB
c-Fos	proteintech	66590-1-IG	1:1000	WB
MKP1	Santa Cruz Biotechnology	sc-373841	1:50~1000	WB, CO-IP
Pan Acetylation	proteintech	66289-1-Ig	1:2000	WB
DDDDK tag	Abcam	ab205606	1:30	CO-IP
GST tag	Abcam	ab9085	1:1500	WB
6×His antibody	Abcam	ab1187	1:4000	WB

Supplementary Table S2. Primers used in the present study

Gene	Species	Forward primer (5→3)	Reverse primer (5→3)
HDAC3	Mouse	CCCAGACTTCACACTTCATCCA	GGTCCAGATACTGGCGTGAGTT
HDAC3	Human	TGGACGTGGAAGAGGTGTATTGA	CCGTAGATGCTCCAGCATTGA
GAPDH	Mouse	ACTCCACTCACGGCAAATTC	TCTCCTATGGTGGTGACGACA
GAPDH	Human	GCCCTCAACGACCACTTTGT	TGGTGGTCCAGGGGTCTTAC
IL-6	Mouse	CCCCAATTTCCAATGCTCTCC	CGCACTAGGTTTGCCGAGTA
TNF-α	Mouse	ACTGAACTTCGGGGTGATCGGT	TGGTTTGCTACGACGTGGGCTA
IL-1β	Mouse	AATGAAGGAACGGAGGAGCC	CTCCAGCCAAGCTTCCTTGT
IL-6	Human	ATGAGGAGACTTGCCTGGTGAA	CTCTGGCTTGTTCCTCACTACTCTC
TNF-α	Human	GCTGCACTTTGGAGTGATCG	ATGAGGTACAGGCCCTCTGA
IL-1β	Human	TACCTGTCCTGCGTGTTGAAA	GGTGCTGATGTACCAGTTGGG

Supplementary Table S3. Sequences of small interfering RNAs used in this study

Gene	Species	Sense strand (5′→3′)	Antisense strand (5′→3′)
HDAC3	human	GGUAGUGGACUUCUACCAATT	UUGGUAGAAGUCCACUACCTT
HDAC3	Mouse	CGGUGUUGGACAUAUGAAACA	UUUCAUAUGUCCAACACCGGG
MKP1	human	CGAACAGUGCGCUGAGCUACC	UAGCUCAGCGCACUGUUCGUG
MKP1	Mouse	GGUUUGUAGCUUUCAGUUAUC	UAACUGAAAGCUACAAACCUA
c-Fos	human	GAGGGUUCCUGUAGACCUAGG	UAGGUCUACAGGAACCCUCUA
c-Fos	Mouse	GGUUCAUUAUUGGAAUUAACC	UUAAUUCCAAUAAUGAACCCA

Supplementary Table S4. Plasmids used in this study

Plasmid	Mutation	Vector	Tag / Reporter
Flag-HDAC3	WT	pCMV-3×FLAG-Neo	3×FLAG
Flag-HDAC3	Y298F	pCMV-3×FLAG-Neo	3×FLAG
Flag-HDAC3	N89A	pCMV-3×FLAG-Neo	3×FLAG
Flag-HDAC3	K137A	pCMV-3×FLAG-Neo	3×FLAG
Flag-HDAC3	Y182A	pCMV-3×FLAG-Neo	3×FLAG
Flag-HDAC3	N197A	pCMV-3×FLAG-Neo	3×FLAG
Flag-HDAC3	E212A	pCMV-3×FLAG-Neo	3×FLAG
MKP1	WT	pcDNA3.1(+)	None
MKP1	K57R	pcDNA3.1(+)	None
GST	-	pGEX-6P-1	GST
GST-HDAC3	-	pGEX-6P-1	GST
His-MKP1	-	pET-28a(+)	GST
AP-1 luciferase reporter plasmid	-	pAP-1-Luc Reporter	Firefly luciferase
pGMLR-TK luciferase reporter	-	pGMLR-TK	Renilla luciferase

Supplementary Table S5. Interacting residues of HDAC3 and MKP1 identified by molecular docking analysis.

HDAC3 residue	MKP1 residue	Interaction type	Distance (Å)
Asn89	Arg55	Hydrogen bond	2.98
Lys137	Asp282	Hydrogen bond	3.07、3.226
Tyr182	Ser296	Hydrogen bond	2.92
Tyr182	Lys289	Hydrogen bond	2.55
Asn197	Lys97	Hydrogen bond	2.91
Glu212	Ile294	Hydrogen bond	2.67
Glu140	Lys280	Hydrogen bond	3.83
Pro201	Thr50	Hydrogen bond	3.23
Asp93	Arg54	Hydrogen bond	3.59
Ile428	Arg54	Hydrogen bond	1.27
